# Effect of ACE-inhibition on coronary microvascular function and symptoms in normotensive women with microvascular angina: A randomized placebo-controlled trial

**DOI:** 10.1371/journal.pone.0196962

**Published:** 2018-06-08

**Authors:** Marie Mide Michelsen, Anna Bay Rask, Elena Suhrs, Kristoffer Flintholm Raft, Nis Høst, Eva Prescott

**Affiliations:** Department of Cardiology, Bispebjerg Hospital, University of Copenhagen, Copenhagen, Denmark; Kurume University School of Medicine, JAPAN

## Abstract

**Objective:**

Studies have suggested a beneficial effect of angiotensin-converting enzyme (ACE) inhibition. To explore whether the ACE inhibitor ramipril has a direct effect on the microvasculature beyond the blood pressure (BP) lowering effect, we investigated whether ramipril improved coronary microvascular function in normotensive women with coronary microvascular dysfunction (CMD).

**Methods:**

We included 63 normotensive women with angina, no epicardial stenosis>50% and CMD defined as a coronary flow velocity reserve (CFVR)<2.2 assessed by adenosine stress-echocardiography in a randomized double-blinded, superiority trial with 1:1 allocation to placebo or ramipril (maximum dose 10 mg depending on blood pressure) for 24±6 weeks. Primary outcome was CFVR. Secondary outcomes were left ventricular systolic and diastolic function and symptoms evaluated by Seattle Angina Questionnaire (clinicaltrials.gov, NCT02525081).

**Results:**

Follow-up was available on 55 patients. BP remained unchanged during treatment in both groups. CFVR improved in both the ramipril (p = 0.004) and placebo group (p = 0.026) with no difference between groups (p = 0.63). Symptoms improved in both groups with no significant between-group differences. No changes were detected in parameters of systolic and diastolic function. No serious adverse reactions were reported.

**Conclusions:**

In normotensive women with angina and CMD, treatment with ramipril had no significant effect on CFVR or symptoms compared with placebo. The effect of ACE inhibition previously reported may be mediated by blood pressure reduction.

## Introduction

Angina pectoris in the absence of significant obstructive coronary artery disease (CAD) can be caused by coronary microvascular dysfunction (CMD). CMD causes insufficient coronary blood flow in situations with increased cardiac oxygen demand leading to transient ischemia and pain [1]. Up to 40% of patients with angina and no obstructive CAD have CMD [2–4], which is a predictor of poor cardiovascular prognosis [3]. However, evidence-based treatment strategies are lacking [5].

Vascular remodelling is suggested as a main pathogenic mechanism of CMD in the absence of obstructive CAD [1]. By a reduction in blood pressure, antihypertensive treatment should theoretically increase the blood flow reserve through a reduction in resting coronary flow [6]. However, evidence suggests that treatment with angiotensin-converting enzyme (ACE) inhibitor has an additional beneficial effect. Treatment has been associated with positive vascular changes beyond the antihypertensive effect and has been suggested to improve both non-endothelial and endothelial dependent CMD [7–11]. Concurrently, treatment with ACE-inhibition in patients with refractory microvascular angina has received a IIb recommendation in current guidelines [12]. However, results obtained from interventional studies on the non-endothelial aspect of CMD have been inconsistent [13–26] and whether the effect observed in some studies on coronary microvascular function could be indirectly mediated via treatment of hypertension is unclear.

In salt replete normotensive individuals without heart failure, effect of ACE inhibition on blood pressure is absent or modest [10,27–38]. Therefore, to explore whether the ACE-inhibitor ramipril had a beneficial effect on the coronary microvasculature beyond the blood pressure lowering effect, we designed a 6 months long double blind placebo-controlled study including only normotensive women. Furthermore, to study whether treatment with ACE inhibitor is indicated in patients with angina and normal blood pressure.

## Methods

### Study population

Normotensive women with angina pectoris, no significant obstructive CAD (<50% coronary artery stenosis) and a history of a coronary flow velocity reserve (CFVR)<2.2 assessed by transthoracic Doppler echocardiography (TTDE) with dipyridamole infusion in the iPOWER (ImProve diagnOsis and treatment of Women with angina pEctoris and micRovessel disease) cohort study were systematically invited [4,39,40]. Normal blood pressure was defined as a history of systolic blood pressure ≤150 mmHg and no current treatment for hypertension. Exclusion factors in the iPOWER cohort are described in detail in previous publications [4,39]. In short, participants had no previous history of myocardial infarction, valvular- or congenital heart disease, no severe pulmonary disease and a left ventricular ejection fraction (LVEF) above 45%. Further exclusion factors in this trial were atrial fibrillation, pacemaker, ACE inhibitor or angiotensin II-antagonist treatment, no angina symptoms within 6 months and an estimated glomerular filtration rate (eGFR)<50 mL/min/1.73m^2^. At study baseline examination (initial screening), participants with a baseline CFVR>2.5 indicating no CMD assessed by TTDE with adenosine stress or baseline systolic blood pressure >150 mmHg were excluded.

### Study design

This study is a randomized placebo-controlled two-arm parallel, superiority trial with 1:1 allocation to treatment with the oral ACE inhibitor, ramipril, and an oral matching placebo, as add-on to usual treatment. After baseline measurements participants were randomized in blocks of 10 participants by the pharmacy (Glostrup Apotek, Copenhagen, Denmark). The allocation sequence was concealed in sealed opaque envelopes until the end of the study. Participants, health care providers and data collectors were blinded.

**Fig 1:**
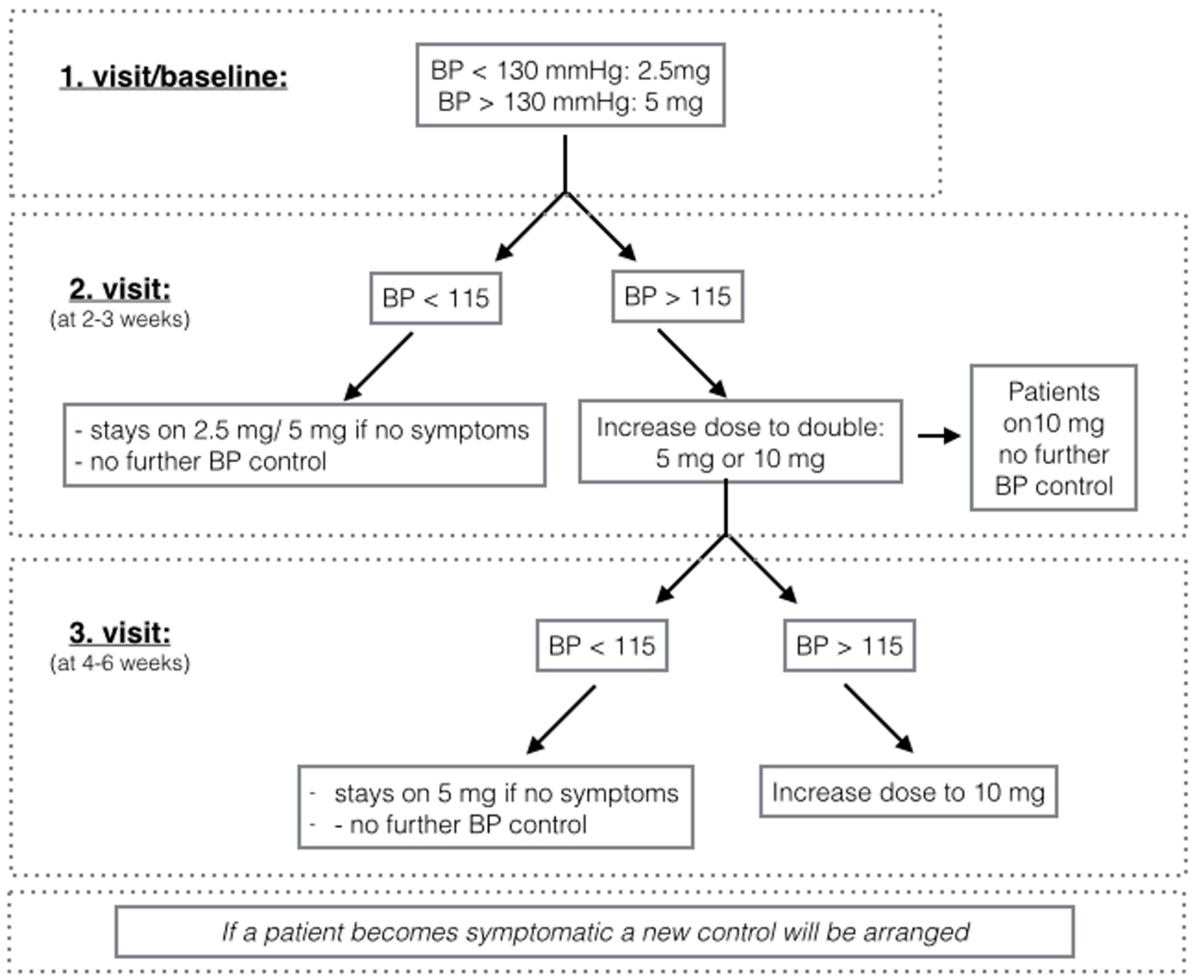
Up titration of project medication. Blood pressure (BP) will be controlled after each visit. If possible treatment dose (per day) is increased. If patients have adverse reactions, project medication dose will be reduced. The least acceptable dose for staying in the study is 2.5 mg per day.

**Fig 2:**
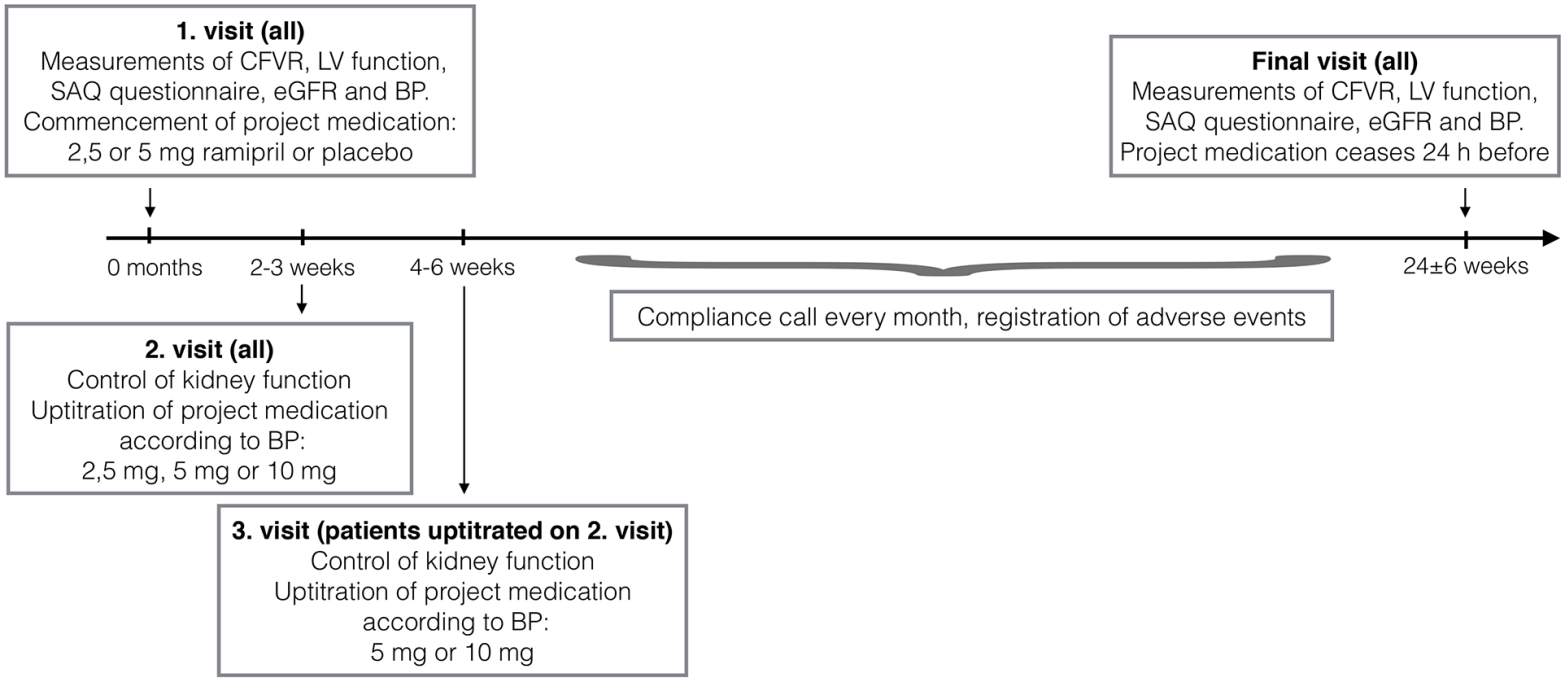
General study outline. CFVR: Coronary flow reserve velocity, LV: Left ventricular, SAQ: Seattle Angina Questionnaire, eGFR: Estimated glomerular filtration rate, BP: blood pressure.

Project medication was up titrated at hospital visits to the highest dose possible according to blood pressure level and side effects following the algorithm depicted in Fig 1. In guidelines or clinical trials the maximum dose of ramipril for treatment of hypertension, heart failure and secondary prevention after myocardial infarction is 10 mg [41–43]. A trial overview is depicted in Fig 2. Kidney function was controlled at each visit by analysing blood samples for creatinine level and estimation of glomerular filtration rate (eGFR). A research assistant, who was not performing the primary endpoint measurements, was in charge of hospital visits and control of adverse events. The pre-specified primary endpoint was CFVR, which was performed by an operator, who had no other contact with the participants. The pre-specified secondary endpoints were the burden of symptoms and parameters of left ventricular systolic and diastolic function measured by TTDE. Examinations were performed at baseline and after 24±6 weeks. The project medication was discontinued 24 hours before the final measurements. After trial commencement no study changes were made.

### Examinations

Assessment included clinical and demographic data. Trained health professionals interviewed participants regarding cardiovascular risk factors including age, body mass index, diabetes, hypertension, and hyperlipidaemia, smoking and family history of cardiovascular disease. Blood pressure and heart rate measures were obtained at rest as the mean of 3 measurements obtained with 3-minute intervals.

Seattle Angina Questionnaire was used to evaluate the burden of symptoms, which were 5 dimensions of functional status: physical limitation, angina stability, angina frequency, treatment satisfaction and disease perception. Participants could score between 0 and 100, and a high score equalled a higher functional status [44].

### Echocardiographic examination

Participants underwent a standard resting transthoracic echocardiography (TTE) using GE Healthcare Vivid E9 cardiovascular ultrasound system (GE Healthcare, Horten, Norway) with a 1.3–4.0 MHz transducer (GE Vivid 5S probe). Images were stored for off-line analysis (GE EchoPAC v.112, Norway). The same experienced echocardiographer, who was blinded to all participant data, performed all image acquisition and analyses at Bispebjerg University Hospital (Copenhagen, Denmark). Before examinations, participants were instructed to be abstinent from caffeine and food containing a significant amount of methylexanthine (coffee, tea, chocolate, cola and banana) for 24 hours. Medication containing dipyridamole was paused for 48 hours, anti-ischemic agents (long-lasting nitroglycerine, beta-blockers, calcium antagonist, ivabradine etc.), anti-hypertensive medication including project medication and diuretics for 24 hours and short-lasting nitroglycerine one hour before the examination. Before the examination, abstinence of the abovementioned food and medication was confirmed.

#### Examination at rest: LVEF, GLS and parameters of diastolic function

We acquired 2-dimensional images of the left ventricle in apical long axis, 2- and 4-chamber views at frame rates between 60–90 frames/s adjusted as close to the patient’s heart rate as possible. Global longitudinal strain (GLS) was measured using software for speckle tracking analysis (Q-analysis, GE EchoPAC v.112, Norway). Aortic valve closure was defined in tissue Doppler M-mode. The left ventricular endocardial border was traced in all three views, and the automatically created region of interest was manually adjusted until tracking was considered optimal. Segments were discarded if tracking was persistently poor. Subsequently, deformation parameters were automatically obtained for all accepted left ventricular segments and GLS was calculated as the average of these [45]. We have previously reported good inter-analyser reproducibility for GLS [39]. In a sub-analysis testing robustness of results, only 3 discarded segments were permitted.

LVEF was analysed as a semi-automated biplane calculation (Auto-EF tool, GE EchoPAC v.112, Norway). Measurements of left ventricular internal dimensions, left ventricle mass index (LVMI), and left atrium volume index (LAVI) by the ‘Volume Method of Discs’ were performed and calculated according to European and American recommendations [46,47]. Echocardiographic parameters of diastolic function including the early (E) and late (A) mitral inflow velocities, tissue Doppler early and late diastolic velocities in the lateral mitral annulus (e’ and a’) and the E/A and e’/a’-ratios were used as surrogates of myocardial relaxation and left ventricular compliance, the deceleration time as a surrogate of early left ventricular stiffness and, E/e’ as a surrogate estimate of left ventricular filling pressures [47]. All measures were averaged over 3 heart cycles. In case of fusion of the E and A or e’ and a’ waves, measurements were registered as missing.

Left ventricular filling pressure was categorized as normal or high by a modified algorithm from the recommendations of the American and European Societies of Echocardiography concerning patients with preserved left ventricular ejection fraction (LVEF), i.e. normal filling pressure if E/e’<8 or E/e’ 8–12 and LAVI<34 mL/m2, and high filling pressure if E/e’ 8–12 and LAVI>34 or E/e’>12, respectively [47].

All parameters were reviewed for outliers by histogram plots and pre-specified cut-offs.

#### Adenosine stress examination: CFVR and left ventricular contractile reserve

TTDE of the left anterior descending artery during rest and adenosine infusion (0.84 mg/kg) over 6 minutes to obtain coronary flow velocities (CFV) at baseline and at maximal hyperaemia using a 2.7–8 MHz transducer (GE Vivid 6S probe) was performed as previously described [4,40]. The primary endpoint, CFVR, was calculated as the ratio between peak diastolic CFV during hyperaemia and during rest. Two experts, blinded to participant data with no participant contact, analysed every CFVR examination independently. The first reading was used, except for estimates that differed by >0.2, in which case the two investigators reanalysed the CFVR examination and reached agreement. In our previous validation study with repeated TTDE CFVR examinations in 10 young, healthy individuals by the same observer, we found an intraclass correlation coefficient (CI) of 0.97 (0.92; 1.00) and limits of agreement (2xSD [CI]) was 0.44 (0.21; 0.68). The test-retest properties were similar in a patient population of 10 women with angina and no obstructive CAD where we found an intraclass correlation coefficient (CI) of 0.90 (0.78; 1.02) and limits of agreement (2xSD [CI]) was 0.48 (0.22; 0.74) [48]. In a sample of 10 participants from the iPOWER study, CFVR readings for the 2 observers were highly reproducible [39]. Two-dimensional images of the left ventricle in apical long axis, 2- and 4-chamber views were acquired at hyperaemia. Strain measurements were analysed as described above. The left ventricular contractile reserve was assessed as an increase of GLS (ΔGLS) from rest to peak hyperaemia.

### Compliance

A research assistant, who was not in charge of the primary endpoint, performed compliance telephone calls each month. Furthermore, we had the following information regarding compliance:

- Participants completed a diary each day indicating whether the daily medication was taken.
- Participants returned excess containers with medication at the end of the study and tablets were counted.

Compliance was defined as the ratio of project medication actually taken and the calculated amount of medication prescribed.

### Statistical analyses

Prior to study commencement, sample size was estimated to be 60 participants completing the study. This was based on the detection of a 0.3 change in CFVR (which we judged as clinically relevant), a power of 90%, and a two–sided significance level of 0.05. We assumed that the SD on mean difference in CFVR would be 0.4 in the ramipril group and 0.3 for the placebo group based on previous studies [48,49]. The intention was to include 72 patients due to expected drop out during the study.

Strict intention-to-treat analysis was not possible due to missing outcomes on participants who dropped out. A linear mixed model with unstructured covariance (PROC MIXED in SAS) was used to perform analysis of the primary (CFVR) and secondary endpoints, including baseline measures on participants who dropped out (analyses were adjusted for baseline value of the outcome studied) [50]. A supplementary analysis investigating the treatment effect on the primary outcome parameter, CFVR, adjusted for resting systolic blood pressure and resting heart rate was performed. Furthermore, a subanalysis stratifying on project medication dosage prescribed was performed. This was not pre-specified in the protocol. We also performed a per protocol analysis including only participants with follow-up completed.

Model control included assumptions of linearity, variance homogeneity, and Gaussian distribution of residuals.

Continuous variables with a Gaussian distribution were expressed as mean ± standard deviation (SD), and continuous variables with a non-Gaussian distribution as median ± interquartile range (IQR). A two-sided p-value below 0.05 was considered significant. All analyses were performed using SAS Enterprise Guide 7.1 (SAS Institute Inc., North Carolina, USA) and STATA/IC 13.1 (StataCorp LP, College Station, Texas, USA).

### Ethical standard

This study was performed in accordance with the Helsinki Declaration and was approved by the Danish Regional Committee on Biomedical Research Ethics (H-3-2014-138) and the Danish Health Authority. Informed consent was obtained from all individual participants included in the study. The clinical trial is registered as ACIM at clinicaltrials.gov (NCT02525081). Flow mediated dilation (FMD) was acquired as part of the original protocol, but data was excluded from the main manuscript due to poor quality of examinations (only 39 patients had a full FMD dataset). Methodology and results are displayed in the Supplementary material (Tables A and B in S1 File). The article is presented according to the CONSORT statement. The authors report no conflicts of interest. All authors have approved the final article.

## Results

### Population

**Fig 3:**
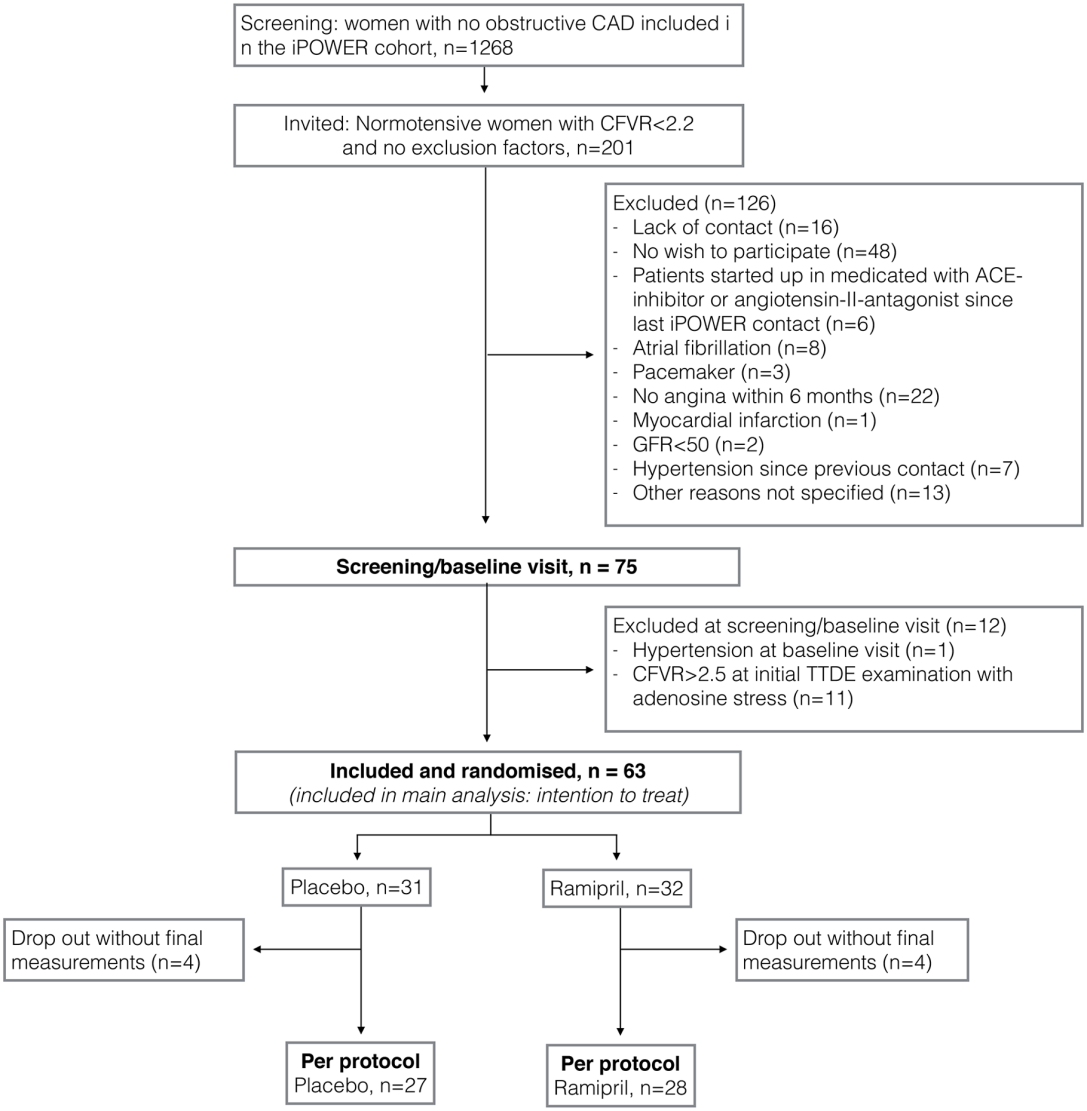
Participant flow-chart. 3 CAD: Coronary artery disease. CFVR: Coronary flow velocity reserve, ACE: Angiotensin-converting enzyme, GFR: Glomerular filtration rate.

A total of 201 cohort participants met the inclusion and exclusion criteria. We included 63 women between the 29^th^ of July 2015 and the 30^th^ of November 2015. Last participant follow-up examination was performed the 28^th^ of April 2016. Eight participants dropped out and final outcome measures were missing as a loss to follow-up: four in the placebo group due to unrelated illness, depression, discomfort and lack of contact and four in the ramipril group due to discomfort, cough and other unrelated illness (Fig 3). Mean age (SD) for the included participants and the participants who dropped out was 58.0 (12.3) and 57.7 (9.9), respectively, and baseline CFVR (SD) was 2.15 (0.32) and 2.11 (0.22), respectively (p>0.05 between included participants and participants that dropped out). Baseline data are displayed in Table 1.

**Table 1:** Baseline characteristics.

	Placebo (n = 31)	Ramipril (n = 32)
Age (years), mean (SD)	57.3 (12.5)	58.6 (11.6)
Body Mass Index, mean (SD)	25.3 (5.4)	27.3 (4.9)
Hypertension, n (%)	0 (0)	0 (0)
Diabetes Mellitus, n (%)	1 (3)	3 (9)
Smoking (current), n (%)	7 (23)	8 (25)
Hypercholesterolaemia, n (%)	12 (39)	19 (59)
Family history of CAD, n (%)	23 (74)	21 (66)
Heart rate (beats/min), mean (SD)	66.2 (10.1)	65.9 (11.3)
Systolic blood pressure (mmHg), mean (SD)	127.4 (11.0)	124.1 (11.0)
Diastolic blood pressure (mmHg), mean (SD)	70.2 (7.7)	67.2 (7.9)
Subclinical atherosclerosis (>0, <50% stenosis at CAG), n (%)	4 (13)	13 (42)
**Medication**		
Acetylsalicylic acid, n (%)	4 (13)	12 (38)
Beta-receptor blockers, n (%)	5 (16)	4 (13)
Statin, n (%)	8 (26)	11 (34)
Calcium antagonist, n (%)	1 (3)	3 (9)
Diuretic, n (%)	3 (10)	5 (16)
**Project relevant information**		
Dosage*		
2.5 mg, n (%)	11 (41)	12 (43)
5 mg, n (%)	4 (15)	6 (21)
10 mg, n (%)	12 (44)	10 (36)
Treatment interval (days)*, mean (SD)	138 (20)	145 (15)
**Echocardiographic parameters**		
CFVR, mean (SD)	2.26 (0.26)	2.03 (0.31)
GLS at rest (%), mean (SD)	-20.4 (2.5)	-21.0 (2.0)
GLS at hyperaemia (%), mean (SD) †	-23.1 (2.6)	-23.5 (2.3)
ΔGLS (%), mean (SD) †	-2.9 (2.3)	-2.5 (2.3)
LVEF (%), mean (SD)	52.6 (4.2)	52.0 (4.7)
LVMI (mg/m^2^), mean (SD)	70.1 (9.8)	72.7 (14.7)
LAI (mL/m^2^), mean (SD)	26.0 (4.5)	28.0 (8.9)
Deceleration time (ms), mean (SD) †	186.0 (27.9)	198.2 (39.2)
E/A ratio, mean (SD) †	1.02 (0.25)	1.04 (0.32)
e’ (cm/s), mean (SD) †	10.21 (2.85)	10.09 (2.57)
E/e’ ratio, mean (SD) †	6.60 (1.53)	7.03 (1.30)
**Burden of symptoms: score by Seattle Angina Questionnaire** ‡		
Physical limitation, mean (SD)	75.79 (17.46)	74.87 (17.69)
Angina stability, mean (SD)	49.66 (29.58)	51.61 (28.18)
Angina frequency, mean (SD)	65.52 (23.24)	78.44 (20.18)
Treatment satisfaction, mean (SD)	69.79 (23.59)	69.15 (5.71)
Perception/quality of life, mean (SD)	54.17 (19.61)	59.01 (23.52)

CFVR: Coronary flow velocity reserve, GLS: Global longitudinal strain, LVEF: Left ventricular ejection fraction, LVMI: Left ventricular mass index, LAI: Left atrium volume (method of discs) index,

*Only including patients that completed the study.

^†^A maximum of 6 observations missing (balanced).

^‡^ A maximum of 8 responses missing (balanced).

### Uptitration and adherence to project medication

Treatment interval (SD) was 138 (20) and 145 (15) days in the placebo and ramipril group, respectively. Approximately 40% of participants were prescribed low dose ramipril (2.5mg), 20% were prescribed middle dose (5mg) and 40% high dose ramipril (10mg) (Table 1).

One participant from the placebo group was terminated early after 50 days of treatment and was not fully up titrated due to discomfort. Further, one participant from the placebo and one from the ramipril group were not fully up titrated according to blood pressure due to presumed adverse reactions.

Counting medication left in project medication containers together with patient diary data, median compliance for patients in the placebo and ramipril group who completed the study was 97% (IQR 93–98%; min. 85%; max. 100%) and 99% (IQR 98–100%; min. 77%; max 100%), respectively.

### Haemodynamics and GFR

Treatment with ramipril did not significantly reduce systolic or diastolic blood pressure compared with placebo (p = 0.91 and p = 0.83, respectively). Heart rate and eGFR did not change during treatment with ramipril compared with placebo (p = 0.09 and p = 0.60, respectively) (Table 2).

**Table 2:** Effect on hemodynamic variables and eGFR.

	Placebo (n = 31)		Ramipril (n = 32)		
	Estimated change at 2. visit (95% CI)	Estimated change at final visit (95% CI)	Estimated change at 2. visit (95% CI)	Estimated change at final visit (95% CI)	p-value *
Systolic BP (mmHg)	-3.4 (-7.2; 0.3)	-0.03 (-3.2; 3.1)	-4.1 (-7.7; -0.5)	0.47 (-2.6; 3.5)	0.91
Diastolic BP (mmHg)	3.7 (0.4; 7.1)	1.5 (-1.0; 4.1)	2.3 (-0.9; 5.6)	1.2 (-1.3; 3.7)	0.83
Heart rate (beats/min)	3.6 (0.9; 6.3)	2.2 (-0.6; 4.9)	-0.2 (-2.8; 2.4)	1.7 (-1.0; 4.4)	0.09
eGFR (mL/min/1.73m^2^)	-2.2 (-4.2; -0.2)	-1.3 (-4.5; 1.9)	-2.6 (-4.6; -0.7)	-3.6 (-6.7; -0.4)	0.60

*p-value obtained by baseline adjusted repeated measure analysis (mixed model)—between group change.

2. Visit at 2–3 weeks after baseline visit. eGFR: estimated glomerular filtration rate.

BP: blood pressure

### Primary endpoint: Effect on CFVR

CFVR improved for both participants receiving ramipril (p = 0.004) and placebo (p = 0.026) with no significant difference in response between the two groups (p = 0.63) (Table 3). Multivariable adjustment with resting systolic blood pressure and resting heart rate did not change results. In a sub-analysis stratifying on final dose of project medication, results obtained for participants receiving middle to high dose ramipril (n = 32) were similar to the overall results with no between-group differences (p-interaction = 0.21).

**Table 3:** Effect of intervention.

	Placebo (n = 31)		Ramipril (n = 32)		
	Estimated Change (95% CI)	p *	Estimated Change (95% CI)	p *	p **
**Echocardiographic parameters**					
CFVR	0.26 (0.03; 0.48)	**0.026**	0.34 (0.11; 0.56)	**0.004**	0.63
GLS at rest (%) †	-0.03 (-0.85; 0. 79)	0.94	-0.24 (-1.07; 0.59)	0.56	0.71
GLS at hyperaemia (%) †	-0.03 (-0.91; 0.86)	0.95	-0.17 (-1.06; 0.72)	0.70	0.80
ΔGLS (%) †	-0.07 (-1.07; 0.93)	0.89	-0.03 (-1.04; 0.99)	0.96	0.94
LVEF (%)	0.93 (-1.14; 3.00)	0.37	0.56 (-1.47; 2.59)	0.59	0.79
LVMI (g/m^2^)	0.76 (-5.11; 6.64)	0.80	0.76 (-5.01; 6.53)	0.79	1.00
LAI (mL/m^2^)	1.44 (-0.89; 3.78)	0.22	1.49 (-0.80; 3.79)	0.20	0.97
Deceleration time (ms) †	-9.53 (-22.97; 3.90)	0.16	-18.19 (-31.83; -4.55)	**0.01**	0.31
E/A ratio †	-0.01 (-0.07; 0.05)	0.72	-0.04 (-0.10; 0.02)	0.10	0.47
e’ (cm/s) †	0.14 (-0.41; 0.70)	0.61	-0.09 (-0.63; 0.46)	0.75	0.56
E/e’ ratio †	-0.15 (-0.68; 0.38)	0.57	-0.22 (-0.75; 0.32)	0.42	0.86
**Burden of symptoms: score by Seattle Angina Questionnaire** ‡§					
Physical limitation	3.74 (-1.85; 9.33)	0.18	-0.36 (-5.99; 5.28)	0.90	0.31
Angina stability	15.39 (5.20; 25.58)	**0.004**	27.33 (17.37; 37.29)	**<0.001**	0.07
Angina frequency	5.59 (-2.67; 13.85)	0.18	11.95 (3.91; 19.99)	**0.004**	0.23
Treatment satisfaction	2.89 (-5.13; 10.91)	0.47	8.24 (0.79; 15.68)	**0.03**	0.27
Perception/quality of life	2.64 (-4.31; 9.58)	0.45	4.39 (-2.27; 11.06)	0.19	0.71

p-value obtained by baseline adjusted repeated measure analysis (mixed model).

p* within group change.

p** between group change.

CFVR: Coronary flow velocity reserve, GLS: Global longitudinal strain, LVEF: Left ventricular ejection fraction, LVMI: Left ventricular mass index, LAI: Left atrium volume (method of discs) index,

^†^A maximum of 9 observations missing (balanced).

^‡^ A maximum of 19 pre-and post treatment responses missing (balanced).

^§^A high score equals a higher functional status.

Results from per protocol analysis were similar to the intention to treat analysis.

### Secondary endpoints: Effect on other echocardiographic measures and burden of symptoms

Outcome measures are displayed in Table 3. There was no effect of treatment with ramipril on parameters of left ventricular systolic or diastolic function assessed by echocardiography compared with placebo.

Angina stability improved significantly in both the ramipril (p<0.001) and the placebo group (p = 0.004). There was an insignificant trend for a larger improvement of angina stability in participants treated with ramipril compared with placebo (p = 0.07). Angina frequency and treatment satisfaction was significantly higher after treatment with ramipril (p = 0.004 and p = 0.03, respectively), but not when compared with placebo (p = 0.23 and p = 0.27, respectively).

Results obtained by per protocol analyses were similar to the intention to treat analyses.

### Adverse reactions

The proportion of participants experiencing an event categorized as either an adverse event, adverse reaction (common side effects) or serious adverse event was not significantly different between treatment groups: 20% vs. 18% (p = 0.74), 22% vs. 28% (p = 0.33) and 5% vs. 5% (p = 0.80), respectively. None of the participants experienced a serious adverse reaction.

## Discussion

In this randomized double-blinded placebo-controlled trial of normotensive women with angina and CMD, we found no significant improvement of coronary microvascular function assessed by CFVR in participants treated with the ACE inhibitor, ramipril, compared with placebo. Further, no effect of treatment with ramipril on burden of symptoms or parameters of left ventricular diastolic and systolic function compared with placebo was detected.

CFVR increased in both groups. This could be a placebo effect and is also likely to be caused by regression towards the mean because participants were selected into the study based on a low CFVR at study entry. As expected, systolic and diastolic blood pressure did not change significantly for patients treated with ramipril. Based on previous studies in normotensive individuals with diabetes and microalbuminuria or nephropathies, we assumed no or only modest blood pressure reduction. In the Heart Outcomes Prevention Evaluation (HOPE) study, ramipril (10 mg per day) lowered blood pressure only modestly (by 3·3/1·4 mm Hg) in high-risk, mostly normotensive patients. However, most studies have found no change in blood pressure [31–38].

A few previous studies have addressed the effect of ACE inhibitor treatment on non-endothelium dependent CMD. In patients with angina and no obstructive CAD, two randomized placebo-controlled studies of ACE inhibition on coronary microvascular function using intracoronary Doppler flow measurements have been conducted. One study (n = 20) showed a significant effect of 10 mg enalapril during 2 months of treatment. Mean systolic blood pressure (SD) was 136 (±20). Drop out was 18% [13]. The other study (n = 61) did not show an effect of 80 mg quinapril over 4 months, but the trial reported an effect in a subanalysis, which was not pre-specified in the trial protocol, of patients with a baseline CFVR<2.5 (n = 32). Approximately 40% had hypertension and mean systolic blood pressure was 126 (±19). Drop out was 21% [14]. In a randomized study including patients with CAD (n = 29), no effect of 6 months of treatment with 40 mg quinapril was found on CFVR assessed by intracoronary Doppler in a non-stenotic target coronary artery compared with placebo. However, only 13 out of 29 randomized patients completed the study [21]. In a randomized comparator study including patients with diabetes (n = 24) CFVR assessed by TTDE improved in the group treated with ACE inhibitor, but the effect was not compared with placebo [17]. Other randomized studies with a comparator have investigated the effect of treatment with ACE inhibitor on coronary microvascular function using positron emission tomography (PET) in patients with hypertension. None of the studies were placebo-controlled and results were inconsistent: One study observed an improvement in coronary microvascular function in the group treated with ACE inhibitor (n = 9) [15], whereas no effect (n = 10, 15) was detected in two of the studies [16,18]. Many small non-randomized studies (n<24) have been conducted using different methods for assessment of coronary microvascular function. In 2 out of 4 small studies including hypertensive patients, ACE inhibitor treatment improved coronary microvascular function [19,20,22,23]. Further, ACE inhibitors in combination with either a calcium antagonist or indapamide have been shown to be beneficial [24,25]. A small study including patients with diabetes also showed an effect [26].

Whether the effect of ACE-inhibition on coronary microvascular function in some studies is indirectly mediated via treatment of hypertension with no direct effect on the microvasculature is uncertain. In support of this, a study found that blood pressure at follow-up after ACE inhibitor treatment could predict change in CFVR [14]. To our knowledge, no previous studies investigating coronary microvascular function have focused exclusively on normotensive patients and contrary to other studies we did not detect an effect compared with placebo. Therefore, this trial suggests that the effect of ACE inhibition on coronary microvascular function previously reported could be mediated by blood pressure reduction and that treatment with ACE inhibition per se does not improve microvascular function.

Studies in patients with angina and no CAD, but a positive stress test or microvascular angina (CFVR<3) have shown that patients treated with a combination of atorvastatin and ramipril or quinapril alone, respectively, significantly reduced burden of symptoms compared with placebo [13,14,51]. In another study of patients with angina, a positive stress test and no obstructive CAD, burden of symptoms were reduced after treatment with enalapril, but it is not mentioned whether this reduction is significant when comparing with the placebo group [13]. In this present study we detected an improvement in the burden of symptoms with ACE inhibitor treatment, which was not significant when comparing with placebo treatment.

A meta-analysis (39 arms, 1068 patients) has shown that ACE inhibitor treatment reduced estimated left ventricular mass by a mean of 10% (95% CI 8%; 12%) [52]. However, the studies included were on patients with hypertension and a more abnormal baseline echocardiography. Similar to our trial, a study of normotensive patients (n = 46) found no change in LVMI assessed by TTDE [53]. Furthermore, most participants in this present trial had echocardiographic parameters of left ventricular diastolic function and LVMI within reference range.

### Strengths and limitations

The study is a randomized placebo-controlled trial. The participants were included systematically, and the study is therefore generalizable for normotensive women with CMD and angina and no obstructive CAD. The primary outcome measure, TTDE CFVR, showed good reproducibility in both healthy individuals and women with angina an no obstructive CAD in our clinic, and the method for CFVR measurements is accessible and highly feasible [39,40,48]. Differences in resting baseline flow will be reflected in CFVR, i.e. an impaired CFVR may be due to a high baseline flow. Therefore, part of the variability in the outcome measure can be due to changes in baseline flow during the trial. However, none of the participants developed disease states known to influence resting blood flow during the trial such as diabetes or hypertension and furthermore we found no significant difference between changes in hemodynamic variables, which are also known to influence resting blood flow, in the group randomized to ACE inhibitors compared with the placebo group.

We intended to include 72 patients to obtain 60 patients completing the study based on a sample size calculation with 90% power. However, only 63 patients could be included from the cohort due to a lower inclusion rate than expected, but 55 patients completed the study with both baseline and follow up measurements (lacking only 5 patients from the original power calculation). The SDs on mean CFVR change obtained in this study were larger than hypothesized probably due to a long treatment interval and a divergent treatment response in the ramipril group (SD 0.4 and 0.7 in the placebo- and ramipril group, respectively). This implies that the change in CFVR needs to be more than 0.4 greater in the ramipril group than the placebo group to detect a significant treatment effect (power set at 80%). Thus, we cannot exclude that we have overlooked a small but clinically relevant effect of the intervention. The two previous studies of ACE-inhibition in patients with no obstructive CAD detected an improvement in CFVR of 0.19 and 1.02 in the intervention group compared with the placebo group, respectively [13,14]. A 13% drop out in this study meets the less than 20% drop out trial quality criteria.

It could be speculated that a higher dosage might have been more effective in yielding persistent coronary microvascular changes. In this present trial the dose of ramipril ranged from 2.5 to 10.0 mg. Due to lower blood pressure at baseline (<115 mmHg) approximately 40% of patients ended up on low dose ramipril, whereas 20% were on medium dose ramipril and 40% on high dose. This was according to the algorithm depicted in Fig 1. However, the maximum dose in the studies investigating the effect of ACE-inhibitor on CMD varies and there seems to be no trend of greater effect on coronary microvascular function with higher dosage. In addition, in the present study no effect of ramipril was detected in subgroups when stratifying by achieved dosage. However, this study was not powered sufficiently to detect effect in subgroups nor was this analysis pre-specified.

Treatment interval was set to 24±6 weeks. Some previous studies looking at coronary microvascular function as a main endpoint found an effect of treatment with ACE-inhibitors between 2–4 months [13,14]. However, the effect seen in these studies may be due to the blood pressure lowering effect of ACE inhibitors. It is possible that a longer treatment period is necessary to result in structural remodelling leading to changes in microvascular function beyond the blood pressure effect.

## Conclusion

Treatment with ACE inhibitor had no significant effect on coronary microvascular function, echocardiographic parameters of systolic and diastolic function or burden of symptoms compared with placebo in normotensive women with angina and CMD. Thus, current guideline recommendations of ACE inhibition in treatment of patients with microvascular angina do not extend to normotensive patients.

## Perspectives

Evidence-based treatment options for patients with angina pectoris are lacking. We investigated the efficacy of treatment with the ACE inhibitor ramipril on coronary microvascular function in normotensive patients with CMD and found no effect compared with placebo, suggesting that the effect previously detected with ACE-inhibition could be mediated through blood pressure reduction. This signifies that treatment with ACE inhibitor might only be indicated for the part of angina patients with hypertension. This could be a step towards a more individualized approach in treating CMD.

## Supporting information

S1 File Supplementary. (DOCX)

S2 File CONSORT 2010 checklist. (PDF)

S3 File Protocol in Danish. (DOCX)

S4 File Protocol in English. (DOCX)

S5 File Data file—Inclusion status. (XLSX)

S6 File Data file—Baseline characteristics. (XLS)

S7 File Data file—Echocardiography data. (XLSX)

S8 File Data file—Echocardiography, strain data. (XLSX)

S9 File Data file—FMD data. (XLSX)

S10 File Data file—Burden of symptoms. (XLSX)

S11 File Data file—Adverse events. (XLS)

## Abbreviations

ACE: Angiotensin-converting enzyme
BP: Blood pressure
CAD: Coronary artery disease
CFV: Coronary flow velocity
CFVR: Coronary flow velocity reserve
CMD: Coronary microvascular dysfunction
eGFR: Estimated glomerular filtration rate
FEV1: Forced expiratory volume in 1 second
GLS: Global longitudinal strain
iPOWER: ImProve diagnOsis and treatment of Women with angina pEctoris and micRovessel disease
IQR: Interquartile range
LAI: Left atrium index
LV: Left ventricle
LVEF: Left ventricular ejection fraction
LVMI: Left ventricle mass index
SD: Standard deviation
TTDE: Transthoracic Doppler echocardiography
ΔGLS: Absolute increase of global longitudinal strain
ΔLVEF: Absolute increase of left ventricular ejection fraction
