# Supplementary material for: Effect of ACE-inhibition on coronary microvascular function and symptoms in normotensive women with microvascular angina: A randomized placebo-controlled trial
Source: PLoS One. 2018 Jun 8;13(6):e0196962. doi: 10.1371/journal.pone.0196962 (PMC5993253; doi:10.1371/journal.pone.0196962)
Supplement: S1 File — (DOCX) [file pone.0196962.s001.docx]

**Supplementary**

**Flow-mediated dilation examination and analysis**

Endothelial function was measured by flow-mediated dilation (FMD) according to guidelines [26] in a standardized setting (i.e., a quiet, dark, temperate environment [21°C to 24°C]) with a GE Healthcare Vivid E9 ultrasound system using a linear array transducer (9L-D probe, GE Healthcare, Horten, Norway) by a research assistant. Participants were fasting for 6 hours before examination. The participants were resting in a supine position for at least 20 minutes prior to the exam.

The ultrasound transducer was placed parallel to the brachial artery 3-5 centimetres proximally of the antecubital fossa and fixed when obtaining a high quality image using a custom made stereotactic probe-holding device. A pneumatic cuff (D.E. Hokanson, Bellevue, WA, USA) was placed distally to the antecubital fossa around the forearm.

Resting arterial diameter was recorded continuously for 1 minute prior to arterial occlusion, which was obtained by rapid cuff inflation to occlusion at 300 mmHg for 5 minutes. Arterial flow and diameter of the brachial artery were recorded continuously 30 seconds before rapid cuff deflation and 4.5 during hyperaemia. Participants were resting for a minimum of 15 minutes to ensure normal vascular conditions. Subsequently, for the nitroglycerine-mediated dilation (NMD), 0.4 mg of nitroglycerine was administered sublingually and arterial flow and diameter of the brachial artery were recorded throughout 8 minutes.

An analyst that had no contact with participants and was blinded to all participant data analysed FMD recordings. Image analyses were performed offline using automatic edge-detection software (Vascular Tools 6, MIA, LLC, IA, USA). The arterial diameters were measured as an average over 5 seconds. FMD was determined as the ratio between peak arterial diameter and resting arterial diameter during reactive hyperaemia. FMD, NMD, the shear rate area under the curve to peak and the resting and peak arterial diameter were reported according to guidelines (1).

The analyst evaluated the quality of ultrasound recordings and excluded those of poor quality.

### Results on flow mediated dilation

A full dataset was obtained for only 39 patients, as 16 patients (29.1%) were eliminated before the final analysis due to a saving error on the ultrasound system (n = 5) and poor image quality making analysis impossible (n=11). Baseline descriptives are displayed in Supplementary Table 1 and outcome measures are displayed in Supplementary Table 2. There was no effect of treatment with ramipril on FMD or NMD compared with placebo. There was a within group change of arterial diameter in the ramipril group, but no effect compared with placebo.

Results obtained by per protocol analyses were similar to the intention to treat analyses.

***Supplementary Table A: Baseline characteristics***

|  | **Placebo (n=31)** | **Ramipril (n=32)** |
| --- | --- | --- |
| FMD (%), mean (SD) | 9.27 (4.88) | 9.26 (5.19) |
| NMD (%), mean (SD) | 26.26 (8.20) | 27.27 (8.84) |
| Resting arterial diameter (mm), mean (SD) | 3.36 (0.56) | 3.32 (0.44) |
| Arterial diameter at peak hyperaemia (mm), mean (SD) | 3.61 (0.60) | 3.59 (0.38) |
| Shear rate area under the curve to peak (s^-1^×s), mean (SD) | 15645 (6999) | 22018 (11941) |

Legend: FMD: Flow-mediated dilation, NMD: Nitroglycerine-mediated dilation. § A maximum of 20 observations missing (balanced).

***Supplementary Table B: Effect of intervention***

|  | Placebo (n=31) | |  | Ramipril (n=32) | | |
| --- | --- | --- | --- | --- | --- | --- |
|  | Estimated Change (95% CI) | p * |  | Estimated Change (95% CI) | p * | p ** |
| **Flow-mediated dilation analysis,** § |  |  |  |  |  |  |
| FMD (%), mean (SD) | 0.77 (-1.50; 3.03) | 0.50 |  | 0.40 (-1.81; 2.61) | 0.72 | 0.77 |
| NMD (%), mean (SD) | -2.21 (-6.01; 1.59) | 0.25 |  | -2.21 (-5.94; 1.52) | 0.24 | 1.00 |
| Resting arterial diameter (mm), mean (SD) | -0.07 (-0.23; 0.08) | 0.35 |  | -0.20 (-0.35; -0.06) | **0.008** | 0.20 |
| Arterial diameter at peak hyperaemia (mm), mean (SD) | -0.06 (-0.24; 0.11) | 0.47 |  | -0.20 (-0.36; -0.03) | **0.02** | 0.24 |
| shear rate area under the curve to peak (s^-1^ ×s), mean (SD) | 235 (-5100; 5569) | 0.93 |  | 5049 (-58; 10157) | **0.05** | 0.14 |

p-value obtained by baseline adjusted repeated measure analysis (mixed model). p* within group change. p** between group change. FMD: Flow-mediated dilation, NMD: Nitroglycerine-mediated dilation § A maximum of 32 observations missing (balanced).

**Reference**

1. Thijssen DHJ, Black MA, Pyke KE, Padilla J, Atkinson G, Harris RA, et al. Assessment of flow-mediated dilation in humans: a methodological and physiological guideline. Am J Physiol Heart Circ Physiol. 2011 Jan;300(1):H2-12.
