# Supplementary material for: Effect of ACE-inhibition on coronary microvascular function and symptoms in normotensive women with microvascular angina: A randomized placebo-controlled trial
Source: PLoS One. 2018 Jun 8;13(6):e0196962. doi: 10.1371/journal.pone.0196962 (PMC5993253; doi:10.1371/journal.pone.0196962)
Supplement: S3 File — (DOCX) [file pone.0196962.s003.docx]

###### Protokol endelig version, 29/6 2015

# Forside

**Titel:** Effect of ACE-Inhibition on Microvascular Function in Women with Assessed Microvascular Dysfunction and No Obstructive Coronary Artery Disease.

**Kort titel:** ACIM

**EudraCT nr: 2014-004490-17**

**Version:** endelig protokol – Godkendete protokoller fra VEK og SST er samlet i et dokument

**Dato:** 29.06.2015

**Underskrift**


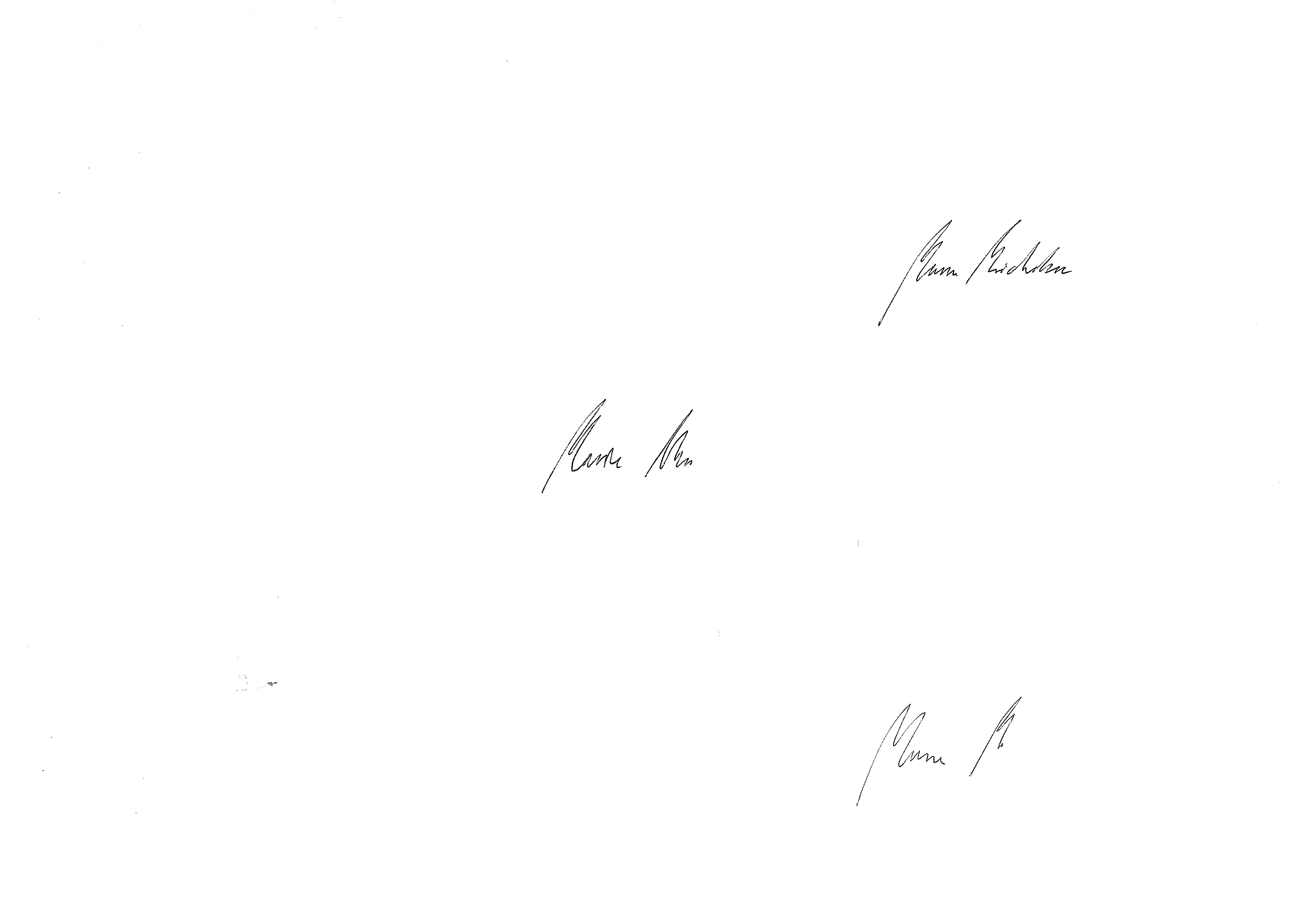
Marie Michelsen

Læge, ph.d.-studerende

og


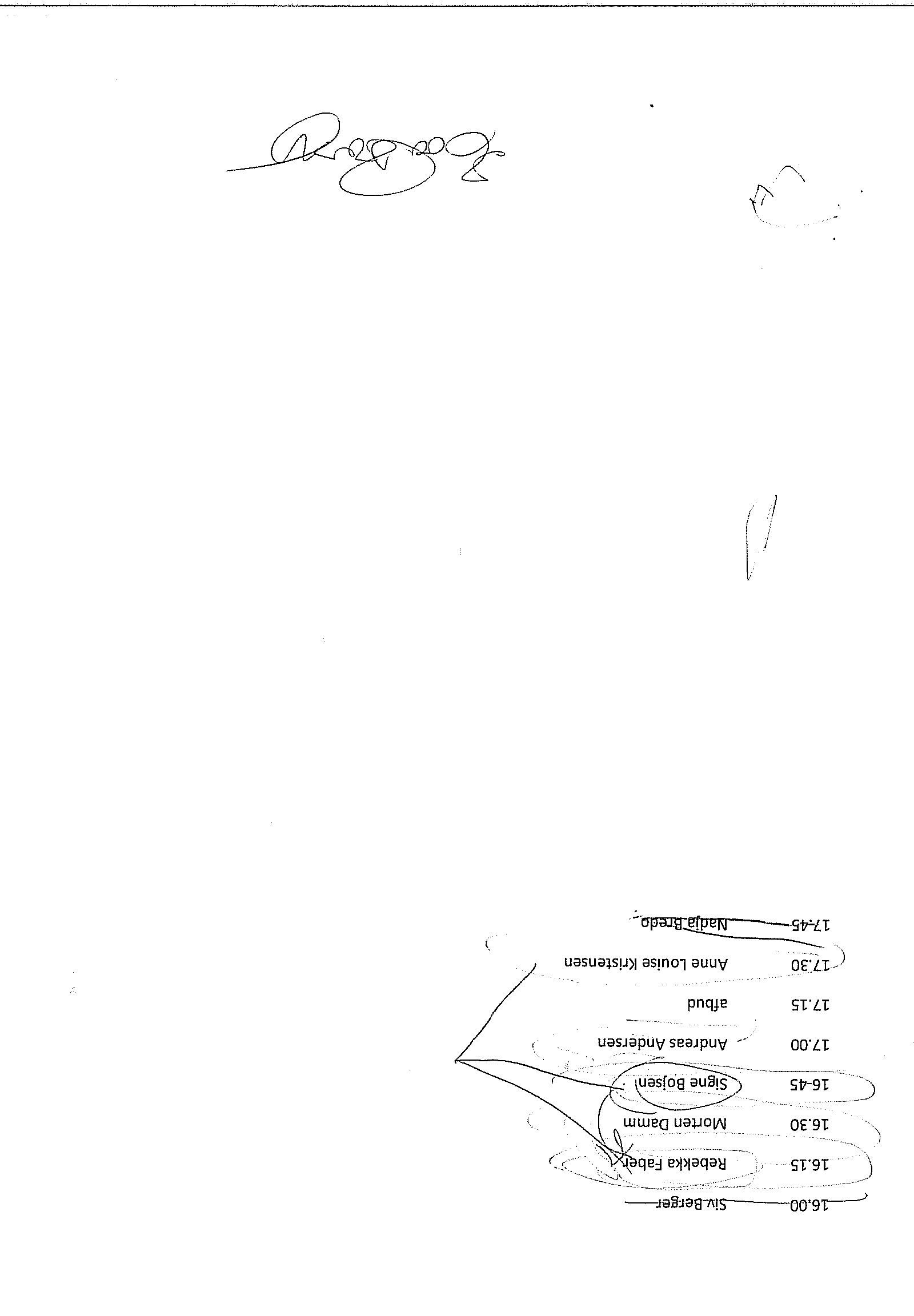
Eva Prescott

Professor, overlæge

## Generelle oplysninger

Protocol Title: Effect of ACE-Inhibitor on Microvascular Myocardial Function in Women with Assessed Microvascular Dysfunction and No Obstructive Coronary Artery Disease.

Protocol code number: 72

EudraCT-nummer: **2014-004490-17**

##### Bispebjerg 29-06-2015

Sponsor:

Professor Eva Prescott autorisations ID: 00MWC

Kardiologisk afdeling Y, Bispebjerg Hospital, Bispebjerg Bakke 23, 2400 Kbh NV

Investigator:

MD Marie Michelsen autorisations ID: 08LPN

Kardiologisk afdeling Y, Bispebjerg Hospital, Bispebjerg Bakke 23, 2400 Kbh NV

*Forsøget udføres i overensstemmelse med nedenstående protokol, ICH GCP og gældende myndigheders regler. GCP-enheden, Region Hovedstaden ved Anne Mette Budde monitorerer dette projekt.*

Tidsplan:

Juli 2015 –januar 2016: Inklusion og randomisering.

Juli 2015 –december 2016: Forsøgsperioden.

**Første patients første besøg: 01.07.2015**

**Sidste patients sidste besøg: 31.12.2016**

**Medicin**

Forsøgslægemiddel: ACE-inhibitor (ramipril)

Værktøjsmedicin: Dipyridamol/adenosin, nitroglycerin

Rescue medicin: Teophyllin

*Reference dokumenter er vedlagt som bilag.*

Table of Contents

Forside 1

Generelle oplysninger 2

Forkortelser 4

Baggrundsinformation om lægemidlet 4

Background 4

Aim 5

Hypothesis 5

Design of the study 5

Endpoints 6

Spin-off study 6

Research design 6

Randomization procedure 7

Trial medicin and blinding 7

Source data 9

Research subjects 9

Inclusion criteria 9

Exclusion criteria 9

Withdrawal criteria 10

Statistical considerations 11

Out come measures and their justification 11

Echocardiography 11

Endothelial function 12

Blood samples 12

Risks and security 12

Medication 12

Coronary flow reserve 12

Endothelial function 13

Blood samples 13

Bivirkninger/Hændelser 13

Direkte adgang til data 14

Kvalitetskontrol og kvalitetssikring 14

Forløb for indhentelse af informeret samtykke 14

Håndtering og arkivering af data 14

Finansiering og forsikring 14

Retningslinjer for publikation 14

Etiske spørgsmål 15

Gennemførlighed 15

Reference liste 15

Annex 1: medicinudlevering 19

## Forkortelser

ACE inhibitor: angiotensin-converting-enzyme inhibitor

CFR: Coronary Flow reserve

FMD: Flow mediated dilation

NO-CAD: No Obstructive Coronary Artery Disease

CAD: Coronary artery disease

TTDE: transthoracic Doppler echocardiography

BP: blood pressure

## Baggrundsinformation om lægemidlet

Der anvendes Ramipril®, som er godkendt til behandling af hypertension og hjerteinsufficiens. Flere studier tyder på, at ACE-hæmmer kan have gavnlig effekt på nedsat funktion af de små kar. Der henvises venligst til nedenstående baggrundsafsnit.

Behandlingen tåles som regel godt og giver få bivirkninger. Hyppigste bivirkninger er blodtryksfald, nyrepåvirkning, stigning af kalium i blodet samt vedvarende tør irritativ hoste, som ses hos omkring 10% af patienterne. I sjældne tilfælde kan ses angioneurotisk ødem, hvilket kontraindicerer fremtidigt brug af præparatgruppen.

ACE-hæmmer opstartes og optitreres efter gældende retningslinjer til mest anvendte maksimale anvendte dosis (10 mg x 1 daglig) afhængig af blodtryk. Behandlingen kontrolleres og monitoreres ved 1 til 2 ekstra besøg efter baseline besøg. Ved baseline besøg startes med 2,5 mg Ramipril x 1 daglig hos patienter med blodtryk < 130 og 5 mg for patienter med blodtryk > 130. Ved besøg 2 (efter 2-3 uger) øges dosis til det dobbelte hvis blodtrykket ikke er under 115 mmHg systolisk. Ved besøg 3 efter yderligere 2-3 uger øges dosis til 10 mg hvis blodtrykket igen ikke er under 115 mmHg. Når patienter er på deres maksimale dosis enten pga. et blodtryk < 115 mmHg eller 10 mg behandlingsdosis overgår patienter til kontrol ved månedlige opkald (se fig. 1 og 2). Nyrefunktion kontrolleres ved baseline, besøg 2 og besøg 3 for patienter som kommer til 3. Besøg. Vi forventer i henhold til baggrundslitteraturen af se en direkte effekt af ACE-hæmmer på koronar flow reserve (CFR), flow medieret dilation (FMD) samt symptom-lindring. Vi forventer, at en behandlingsperiode på 6±1,5 måneder kan påvise en effekt.

Forsøgspopulationen udgøres af patienter fra en allerede eksisterende kohorte af kvinder med angina pectoris uden stenoser påvist ved koronar arteriografi, iPower-studiet^1^. Der er 111 antal patienter som møder inklusions og eksklusion kriterier pr. 09.05.2014. Inklusion til iPower kohorten er kontinuerlig til start af ACIM-studiet således at denne population fortsat vil vokse inden inklusion til ACIM afsluttes. Der forudses derfor ingen problemer med rekvirering af forsøgspersoner til studiet.

*De følgende afsnit vil være på engelsk*

## Background

Microvascular angina is proposed to be myocardial ischemia caused by microvascular dysfunction. When the microvasculature is dysfunctioning, blood flow in the coronary vessels does not increase sufficiently to meet oxygen demand leading to ischemia and pain. In the absence of stenosis of major coronary arteries, coronary flow reserve (CFR) reflects coronary microcirculation^2^. Up to 50% of patients with angina and no obstructive coronary artery disease (NO-CAD) have impaired CFR, which is a strong predictor of poor cardiovascular prognosis^3–5^. Furthermore CFR has been shown to be associated with conventional risk factors; among these hypertension^6,7^.

ACE-inhibitor treatment of patients with Angina Pectoris and/or essential hypertension and no stenosis on coronary angiogram improves CFR measured invasively by Doppler Guidewire or gas chromotography^8–11^ suggesting an effect of ACE inhibition on the microvasculature. Studies assessing the effect of ACE-inhibition in hypertensive and mild CAD patients with CFR assessed by PET showed divergent results. One study showed an improvement on both resting and hyperaemic myocardial blood flow^12^ , another showed an increased myocardial perfusion reserve post treatment^13^ and a third failed to show any significant effect^14^. If the effect seen on CFR is indirectly mediated via treatment of hypertension or if ACE-inhibition has a direct effect on the microvasculature continues to be uncertain in patients with microvascular angina. Some studies show an effect on CFR in both normotensive and hypertensive patients with microvascular angina^9^ whereas other studies only show an effect in hypertensive patients and not in normotensive^11^. Treating patients with diabetes and a CFR>2.0 with ACE-inhibitor has also proven efficient in increasing CFR measured by transthoracic Doppler echocardiography (TTDE) supporting the theory that ACE-inhibition could have a direct effect on the microvasculature^15^. In animal studies ACE-inhibition has shown capability of preventing adverse vascular remodelling dissociated to its blood pressure lowering effect^16^. ACE inhibitor treatment is thought to be associated with vascular changes in CAD patients^17^. However Clinical trials have shown discrepant results regarding reduction of hard endpoints with ACE inhibition in high-risk CAD patients^18–24^. It remains unclear if ACE-inhibition has a direct effect on the microvasculature in normotensive patients with microvascular angina.

Endothelial dysfunction assessed by Flow-dependent endothelium-mediated dilation (FMD) is associated with cardiovascular risk factors and more prominent in women^25,26^. FMD predicts long term cardiovascular events in healthy subjects^27^. Treatment with most types of ACE-inhibitors improves FMD shown in studies with patients with syndrome X, coronary artery disease, and hypertension and in healthy elderly. However Enalapril treatment did not show an affect on FMD^28–34^.

## Aim

The aim of this study is to explore effects of long term treatment with ACE-inhibitor on the microvasculature and endothelial function assessed by coronary flow reserve (CFR) by transthoracic echocardiography and flow mediated dilation (FMD) in normotensive patients with microvascular dysfunction (CFR<2.2) and Angina Pectoris but NO-CAD.

## Hypothesis

ACE inhibitor treatment reverts microvascular remodelling and endothelial dysfunction in patients with microvascular dysfunction and Angina Pectoris but NO-CAD thereby improving microvascular function and reducing symptoms.

## Design of the study

### Endpoints

Primary endpoint: CFR after treatment with ACE-inhibitor/placebo treatment in 6±1,5 months, assessed by a non-invasive Trans-Thoracic Doppler Echocardiography (TTDE).

Secondary endpoints: 1) Symptoms after 6±1,5 months of treatment with ACE-inhibitors assessed by Seattle angina questionnaire 2) exercise level assessed by iPAQ 3) strain assessed by speckle tracking echocardiography after 6±1,5 months of treatment with ACE-inhibitor 4) Endothelial function by FMD after treatment with ACE inhibitor in 6±1,5 months, assessed by flow mediated dilation of the brachial artery by ultrasound.

### Spin-off study

Reproducibility of CFR measurements <2.2 (from entry in iPower and entry in ACIM) in relation to symptom improvement or worsening in the period between measurements.

### Research design

The study is a randomised double-blinded interventional study. 72 patients with microvascular dysfunction assessed by TTDE as a CFR<2.2 are recruited from the iPower cohort (women with angina but no CAD examined with TTDE CFR), Region Zealand. Patients with hypertension will be excluded from the study.

*The patients will be randomised to group 1 or 2:*

1. Group 1: Oral ACE-inhibitor for 6±1,5 months
2. Group 2: Oral matching placebo for 6±1,5 months

Examinations and measurements will be performed at baseline and after 6±1,5 months. There are furthermore 3 possible blood pressure (BP)/kidney function control visits depending on how many up titrations of treatment medicine are necessary for the individual patient (see fig. 2). Compliance calls and control of adverse events will be made every month and the patient can also contact us with any concern.

Participation in the study is 6±1,5 months. 24 hours before final measurements Ramipril treatment will be discontinued. Ramipril treatment will be add-on to usual treatment.

*
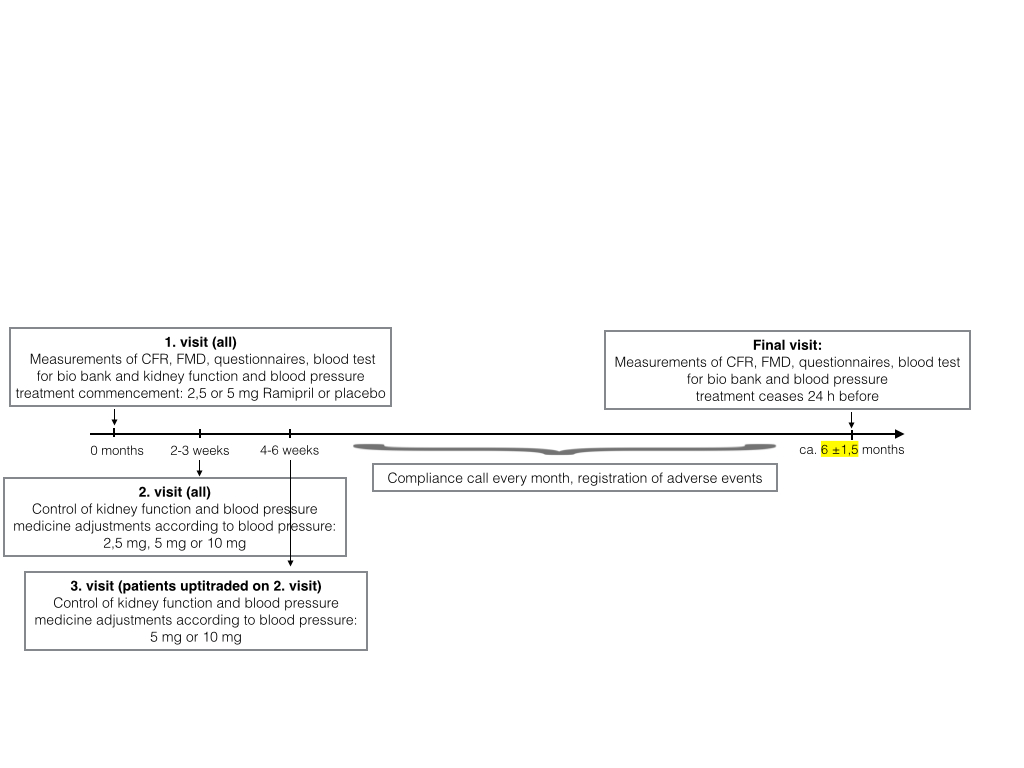
Figure 1: project overview, timeline*


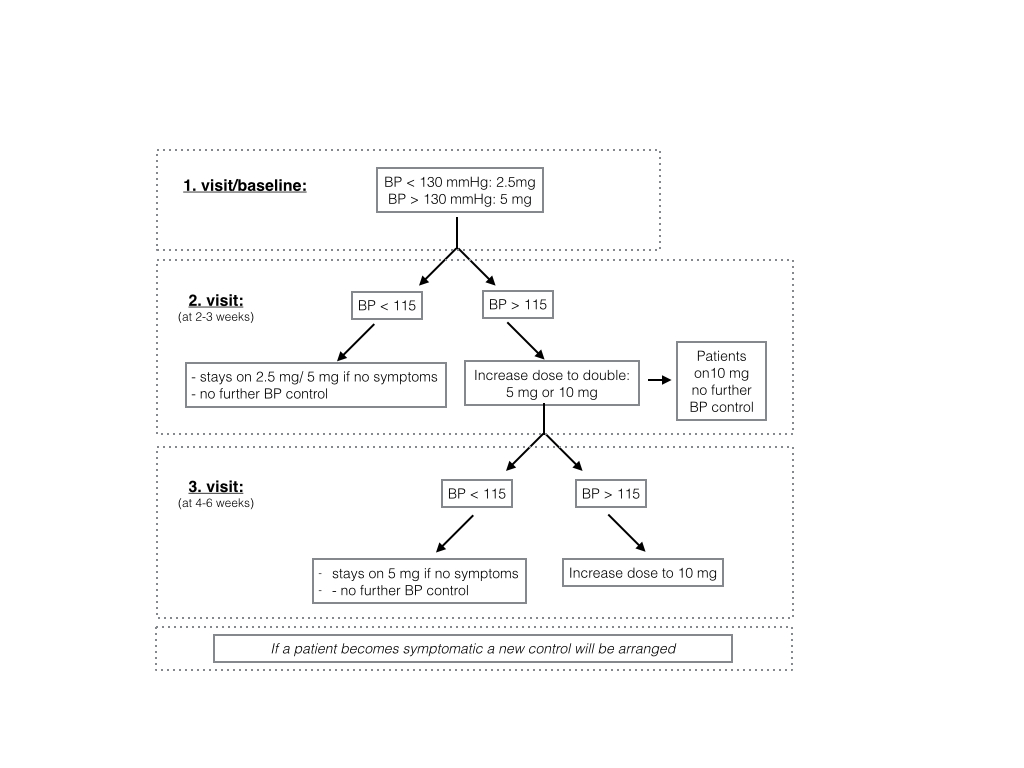


*Figure 2: up titration of Ramipril. Blood pressure (BP) will be controlled after each visit. If possible treatment dose is increased (BP<115) If patients have symptoms of low blood pressure Ramipril dose will be halved or other blood pressure lowering agents will be discontinued. The least acceptable dose for staying in the study is 2.5 mg.*

### Randomization procedure

Patients will be randomized into 2 equal groups receiving either Ramipril or placebo. Randomization is done by the pharmacy (Glostrup Apotek). Id-numbers (ACIM1-72) are allocated equally to either placebo or Ramipril treatment using simple randomization.

Randomization is concealed in a document sealed in an opaque envelope and can only be revealed at the end of the study. For individual patients in occurrence of a serious adverse event the sponsor or investigator will contact the pharmacy to reveal medication type. The sealed envelope will be locked in a cabinet at the office of the sponsor.

The sponsor, investigator, research assistant and patients will have no knowledge of which randomization group the patients belong to

Id-numbers (ACIM1-72) will be allocated to patients in a consecutive order.

### Trial medicin and blinding

Study medication will be prepared by the pharmacy (Glostrup Apotek) in a double-blinded manner. Medication will be delivered in bottles containing 50/100 x 5 mg placebo tablets or 5 mg Ramipril (Ramipril®, Hexal) and both bottle containers and tablets are indistinguishable. Tablets are produced with a pressure sensitive score notch and can be divided into equal halves. Bottle containers will be labelled with a study id-number ACIM1-ACIM72 (identification number/treatment number), the batch and/or code number to identify the contents and packaging operation, the name of the investigator Marie Michelsen/research assistant and mobile number, pharmaceutical dosage form, route of administration, quantity of dosage units, indication of use: “clinical trial”, the storage conditions, period of use (expiry date) and warning label: “keep out of reach of children”. The directions for use will be made to a leaflet according to individual dosage because of different final dosage after up titrations.

In both the placebo and Ramipril group medication will start with either 2,5 mg (BP<130) or 5 mg (BP>130) daily. After 2-3 weeks the dose is doubled to 5 mg or 10 mg unless blood pressure is below 115 mmHg. If blood pressure continues to be higher than 115 mmHg for patients up titrated to 5 mg treatment dose will be doubled to 10 mg at the third visit. Blood pressure and kidney function will be controlled at each visit (see fig. 1). If patients have symptoms of hypotension (dizziness, syncope or high pulse) treatment dose will be reduced and/or other blood pressure lowering treatment discontinued or if necessary Ramipril treatment discontinued.

If oral ACE-inhibitors are not tolerated treatment will be discontinued. Regarding exclusion for treatment discontinuation please see ”withdrawal criteria”.

Patients taking a dose of

- 2,5 mg will take a half tablet a day
- 5 mg will take one whole tablet a day
- 10 mg will take two tablets a day

Patients will be up titrated in same manner independent of which medication they are on. For each study id-number medication for up titration is available. Patients will receive the appropriate amount of bottles according to uptritrated dose level (annex 1).

Patients will be up titrated and monitored by a research assistant so the investigator making the primary endpoint measure is unaware of the final treatment dose. In case of any adverse events/reactions or serious events/reactions the research assistant will confer with the sponsor or a project physician if necessary and not the investigator to avoid awareness of medication type for person obtaining primary endpoint. Only in case of a suspected serious reaction medication type will be unblinded by the sponsor or investigator by calling the pharmacy, Døgnåbent Glostrup Apotek, with patients id/cpr-number. Regarding exclusion for unblinding please see ”withdrawal criteria”.

Analysis of echocardiographic measurements for primary and secondary measurements will be made minimum 1 month after baseline and final visit so the investigator is unaware of blood pressure measurements during examinations.

Control of compliance will be made at visits and hereafter every month by patient structured open questions interviews (visits and telephone) Furthermore patients will make a diary every day of pill intake and any symptoms (potential adverse effects).

At the end of the study the patient will return used pill containers with remaining tablets within. These will be counted again to ensure medication compliance and accuracy.

### Source data

Indication of data and where to find the original source data:

- Allocation of id-number (ACIM) – booking calendar /CRF
- Blood pressure monitoring: blood-pressure notes/directly written in CFR
- Final dose for medication: booking calendar /directly written in CRF
- Kidney function: hospital electronic system (LABKA)
- In/exclusion criteria check: directly written in CRF /patient journal
- Adverse event/reactions: directly written in CRF or patient journal
- Medicine record (pause/compliance): directly written in CRF
- Questionnaires: paper version completed by patients
- Echocardiographic measurements: in worksheet of echopac analysis program

## Research subjects

### Inclusion criteria

Patients from the iPower cohort with microvascular dysfunction defined as a TTDE measured CFR < 2.2 with a good quality (quality index > 3) examination who are normotensive will be included in the study. Patients with a CFR <2.0 will be invited before patients with a CFR between 2.0 - 2.2. Normotensive will in this study be defined as patients who have a blood pressure ≤ 150 at last visit in iPower and who are not in treatment for documented hypertension. Patients will be found searching for patients in the iPower database with these criteria.

The iPower cohort has included women aged 18-80 with angina-like chest discomfort but no obstructive coronary artery disease (Coronary angiography with no significant stenotic lesions (<=50%) of epicardial vessels performed within 1 year of inclusion) since 2012.

For women in the fertile age a negative pregnancy test (urine HCG) and the use of safe anti conception is obligatory. Safe anticonception is regarded as: spiral, birth control, vaginalring, transdermal patches, implant, depot injection, women in a fixed relation with a sterile partner or the use of the double barrier. Anticonception is used for the whole treatment period and 15 days after the last day in the research project.

Sterile and infertile women will not have to use anticonception. Sterility or infertility is defined as bilateral tubectomy, hysterectomy and bilateral ovariectomy. Anticonception will not be used in postmenopausal women regarded as loss of menstruation in more than 12 months.

Inclusion in ACIM will be consecutive until 72 patients have been included. There will be no follow up after the last examination (6±1,5 months). Thereafter, the patients can continue on ACE-inhibitor if they want and follow routine control at their general practitioner. They will be informed that ACE-inhibitor treatment is only paid during participation in the study.

### Exclusion criteria

Exclusion criteria are (ascertained through iPower cohort database, patient records and patient contact)

- Current treatment with ACE-inhibitors or Angiotensin II-antagonists
- Atrial fibrillation
- Pace-maker
- Allergy towards Ace-inhibitor, Ramipril ® or tool-medicine: Dipyridamole/adenosine, Nitro-glycerine or rescue medicine: Theophylline
- Baseline CFR >2.5 when entering ACIM-study.
- No episodes of chest pain within 6 months before inclusion
- Coronary angiography with significant stenotic lesions (>/=50%)
- Other cause of chest discomfort deemed highly likely
- Left ventricular ejection fraction below 45% assessed by echocardiography at baseline measurement
- Significant valvular heart disease (Definition: Verified in medical records after echocardiography. If the echocardiographer in this study suspects valvular heart disease, the patient is referred for expert evaluation and excluded from the study until valvular disease has been excluded. All definitions are taken from the guidelines of the Danish Society of Cardiology (DCS).
  - Haemodynamic significant Aortic Stenosis: Valve area < 1 cm^2^ or <0.6 cm^2^/m^2^ body surface area.
  - Severe aorta Regurgitation (AR): Vena contracta > 6 mm, Moderate/severe LV volume load, ERO > 0.3 cm².
  - Mitral Stenosis (MS): Valve area < 2.5 cm2.
  - Severe Mitral Regurgitation (MR): ERO > 0.4 cm², Moderate/severe LV-load, Vena contracta > 6 mm.
- Congenital heart disease or cardiomyopathy verified in medical records
- Significant co-morbidity with < 1 year expected survival: decision made by the person responsible for inclusion based on the patient interview and/or medical records.
- Severe COPD with FEV1<50% of predicted
- Severe asthma defined as asthma which requires treatment with high dose inhaled corticosteroids (ICS) plus a second controller (long acting β2 agonist (LABA), leukotriene modifier, theophylline or systemic corticosteroids) to prevent it from becoming uncontrolled or which remains uncontrolled despite this therapy."
- Previous verified myocardial infarction (Definition: verified in medical records, STEMI (ST segment elevation, elevated enzymes) or NSTEMI (elevated enzymes, ECG changes/no ECG changes).
- Previous revascularization (PCI or CABG)
- Elevated cardiac biomarkers: Troponin > 50 ng/l (high sensitive) or > 0.03 μg/l (4. generation), CKMB > 4.0 μg/l (women).
- ECG with verified ST-segment elevation
- Language- or other barrier to giving informed consent (for example mental ability to understand project)
- Travel distance: a distance to research hospital requiring more than 3 hours of travel
- Patient unwilling to participate (Low burden of symptoms, other illnesses, “Lack of energy”, transport problems, anxiety because of the examination, other).
- No signed informed consent.
- Other (Pregnancy, significant psychiatric disorder)
- GFR < 50 mL/min/1,73 m^2^

### Withdrawal criteria

Patients who will be withdrawn from participating in the study:

1. Suspected serious reaction where medication type will be unblinded by the sponsor by calling Glostrup pharmacy (open day and night)
2. If they do not want to continue with treatment before total up titration of treatment
3. Sustained side-effects which make the patients unable to take ACE-inhibitor/placebo before total up titration of treatment
4. Poor compliance defined as less than 70 % of the time not taking ACE-inhibitor/placebo assessed by investigator. Patients will also be excluded with a more than 2 months continuous pause from medication or more than 2 weeks continuous pause up to endpoint measurements.
5. Patients who do not wish any endpoint measures
6. GFR < 50 mL/min/1,73 m^2^ and/or 20 % decrease in GFR during commencement of ramipril treatment

If a patient wish to withdraw or have sustained side effects after total up titration of treatment but before finishing 6±1,5 month with more than 70% compliance in the treatment period, endpoint measurements will be performed if patient agree to this and the patient will not be excluded from the study.

Excluded patients will no longer be followed but go to their general practitioner in regard to any health problems as normal.

If there is more than the expected 20% drop out from the study, new subjects will be recruited assuring 60 subjects who obtain final main endpoint measure in the study, if possible.

## Statistical considerations

Statistical test for randomized control study designs will be used.

We hypothesized that the ACE-inhibitor treated group would have a higher CFR at 6±1,5 months compared to placebo. Prior data from iPower show that women meeting the inclusion criteria of the present study had a mean CFR of 1.9 with a standard deviation (SD) of 0.22. A reproducibility study in young healthy volunteers at our clinic with same echocardiographer for the present study had a SD of 0.23 on repeated measurements within 5 days. Furthermore a study (same echocardiographers) with diabetes patients had a SD of 0.32 on repeated measurements and 0.38 on repeated measurements after treatment with GLP1-analogue. We therefore assume that the SD on mean difference in CFR will be 0.4 for the ACE inhibitor populations and 0.3 for the placebo group. An improvement of 0.3 (i.e. approx. 15%) in CFR is regarded as clinically relevant. An estimated sample size of 60 was calculated to be necessary for detection a 0.3 difference in CFR with a power of 90% and a two –sided significance level of 5%. Assuming a 20% dropout rate, enrolment was set at 72 patients to reach 60 completed patients.

## Out come measures and their justification

Following examinations will be performed at baseline and after intervention.

### Echocardiography

Examination will be performed at rest and during dipyridamole/adenosine infusion for CFR measurement. CFR can be reliably assessed non-invasively by trans-thoracic Doppler flow echocardiography of the left anterior descending artery with a good quality (Quality Index>3) in 91 % of the iPower population (abstract accepted at EuroEcho imaging conference). Improvement in cardiac function following intervention may be subtle and is more likely measurable during stress. We will therefore measure and analyse systolic and diastolic function including strain at rest and during pharmacological stress with adenosine/dipyridamole. Examination will be at baseline and after 6±1,5 months and analysis of measurements one month later.

### Endothelial function

Endothelial function will be measured by flow mediated dilation. Brachial artery 2-dimensional and pulsed Doppler flow velocity signals will be obtained above the antecubital crease with a linear array transducer, using a cardiovascular ultrasound system (GE healthcare vivid E9). Hyperaemia is induced by inflating a blood pressure cuff on the proximal portion of the arm to 300 mmHg occluding arterial flow for 5 minutes. Pulsed Doppler recordings and 2-dimensional images will be obtained 60 seconds after cuff deflation (endothelial dependent dilation). After a 15-minute rest period to allow restoration of baseline conditions, non–endothelium- dependent brachial artery dilation will be assessed before and after administration of 0.4 mg sublingual nitro-glycerine (NTG). Examination will be at baseline and after 6±1,5 months and analysis of measurements one month later.

### Blood samples

1) Measurements of kidney function at baseline and 2 weeks follow-up

2) Blood samples for a future research bio bank will be taken at baseline and 6±1,5 months after Ramipril treatment (project bio bank)

The total blood loss from each patient is at baseline maximum 35 ml, Control of kidney function maximun9 ml and at final examination maximum25 ml

## Risks and security

### Medication

ACE-inhibitor, Ramipril is well known for treatment of hypertension and heart failure. In general the treatment is well tolerated and few has side effects. Most common side effects are drop in blood pressure, affection of kidney function and dry irritative cough. If the latter is experienced medication will be discontinued and patient excluded from the study. In rare cases angioneurotic oedema can occur. All patients will be informed about side effects. If a patient develops sustained or severe side effects she will be told to discontinue ACE-inhibitor treatment.

All facilities and skilled personnel necessary for study completion are available. CFR measurements on the same population are formerly accepted by “Den Videnskabsetiske Komité Region Hovedstaden” under the ” I power – bedre diagnostik og behandling af kvinder med angina pectoris og småkarssygdom”. Journal number: H -3-2012-005. The study will be discontinued if safety regarding the study should be changed radically.

### Coronary flow reserve

Adenosine/ dipyridamole is routinely used in clinical practice for SPECT scans (myokardiescintigrafi). Administration of adenosine has several potential side effects. The most common are shortness of breath, drop in blood pressure, flushing, headache, bradycardia and 3^rd^ degree AV blockage (www.medicin.dk). These side effects are generally mild and of short duration because of the very short half time of adenosine (seconds). All side effects are typically remitted approximately 1 minute after infusion has stopped. If the patient experiences more pronounced side effects the infusion is discontinued. Advanced resuscitation equipment and easy access to antidote (theophylline) will always be accessible when examination is conducted. Dipyridamole has similar side effects to adenosine but they are generally milder (www.medicin.dk). However due to longer half-life, an antidote (aminophylline, 120-240 mg) is given intravenously after the coronary flow assessment has been performed.

### Endothelial function

Nitro-glycerine is routinely used in clinical practice. Administration can give mild side affects as headache, dizziness and hypotension.

### Blood samples

All blood samples will be taken under sterile conditions and the risk of infection is minimal. Some discomfort can be related to the needle.

After all examination procedures the patients will be observed in the clinic for 30 minutes before they are allowed to leave the hospital.

Blood for biomarker analysis will be kept in project bio bank.

*Følgende afsnit vil være på dansk*

## Bivirkninger/Hændelser

Sponsor/investigator sikrer, at alle oplysninger om SUSAR, som er dødelige eller livstruende, registreres og indberettes til Sundhedsstyrelsen hurtigst muligt og indenfor 2 døgn efter at Sponsor/investigator bliver vidende om denne.

Senest 14 dage efter indberetningen meddeler sponsor/investigator sundstyrelsen Og Videnskabsetisk Komité alle relevante oplysninger om sponsors/ investigators opfølgning på indberetningen.

Alvorlige bivirkninger, som ikke anses som SUSAR (dvs. ikke er uventede), registreres i CRF og indberettes 1 gang årligt. En alvorlig bivirkning defineres som en bivirkning eller hændelse som uanset dosis resulterer i død, er livstruende, medfører hospitalsindlæggelse eller forlængelse af hospitalsophold, resulterer i betydelig eller vedvarende invaliditet eller uarbejdsdygtighed eller fører til en medfødt anomali eller misdannelse.

AE, AR, SAE og SAR registreres og indberettes i den endelige rapport til sundhedsstyrelsen.

Som reference dokument anvendes produktresumé for Ramipril .

En gang årligt i hele forsøgsperioden skal sponsor udarbejde en liste over alle alvorlige formodede bivirkninger, som er indtruffet i forsøgsperioden, og en rapport om forsøgspersonernes sikkerhed. Liste og rapport skal indsendes til sundhedsstyrelsen og Videnskabs etisk komité.

Registreringer foregår fra inklusionstidspunktet til og med sidste undersøgelse sidste dag.

Patienterne som oplever SUSAR eller anden alvorlig bivirkning følges indtil patients sidste forsøgsdag. Herefter vil patienten overgå til kontrol hos egen læge.

## Direkte adgang til data

Investigator giver tilladelse til at adgang til kildedata til sundhedsstyrelsen, GCP enheden Københavns Universitet, Videnskabsetisk komité samt data tilsynet.

## Kvalitetskontrol og kvalitetssikring

Der vil blive etableret procedurer for kvalitetskontrol og kvalitetssikring. GCP enheden ved Københavns Universitet vil forestå monitoreringen. Der gives tilladelse til audit og inspektion.

### Forløb for indhentelse af informeret samtykke

Patienterne udvælges fra iPower-kohorten på baggrund af inklusion og eksklusionskriterier. Kun de patienter som umiddelbart opfylder kriterierne kontaktes og de som har givet tilladelse til kontakt ved inklusion i iPower. Patienterne kontaktes først per brev med information om projektet og herefter per telefon hvor de igen orienteres om projektet, og hvis de er interesserede aftales tid til mundtlig patientinformation. Der tilsendes skriftlig patientinformation eller den gives med hjem.

Der tilbydes 1-2 dage betænkningstid mellem mundtlig patientinformation og indhentelse af informeret samtykke. Det er projektansvarlige læge som er tilknyttet projektet, som tager den første telefoniske kontakt. Patienterne orienteres om muligheden for en bisidder ved informationssamtalen og dette fremgår ligeledes af den skriftlige patientinformation. Den mundtlige patientinformation foregår på et kontor, som kun anvendes til det formål den pågældende dag og forestås af den projekt ansvarlige læge eller forskningsassistent med investigator i nærheden.

Forsøgspersoner kan til hver tid kontakte investigator eller forskningsassistent på projektet.

## Håndtering og arkivering af data

Hver patient vil få et specifikt projekt nummer ACIM-01 til ACIM72. Alle data på patienten vil blive registreret under dette nummer med angivelse af baseline, besøg 2-4, compliance opkald, 6±1,5 mdr. intervention. Alle data vil blive opbevaret i en patientmappe på et aflåst kontor og i elektronisk CFR. Til opbevaring af blodprøver anvendes en fryser med lås som står på Bispebjerg Hospital.

## Finansiering og forsikring

Professor Eva Prescott Kardiologisk afdeling Y, Bispebjerg Hospital er sponsor til dette projekt. Projektet er finansieret af delvist af hjerteforeningen (fra proejtkpulje på 6 mio) og Københavns Universitet (fra projektpulje til phd-løning på ca 1.5 mio). Der udbetales ikke vederlag til forsøgspersonerne. Der vil fortsat blive søgt non-komercielle midler til finansiering af projektet. Ved ny støtte, vil navn og beløb vil blive efteroplyst til de videnskabsetiske komiteer og forsøgspersoner. Hverken sponsor eller investigator er økonmisk interesseret i projektet.

## Retningslinjer for publikation

Både positive, negative og inkonklusive data vil blive publiceret i videnskabelige artikler, abstracts til kongresser og på hjemmesider ([www.ipowerhjerter.dk](http://www.ipowerhjerter.dk)) eller på [www.clinicaltrial.gov](http://www.clinicaltrials.gov/) og på hjemmesider (www.ipowerhjerter.dk). Resultaterne sendes til sundhedsstyrelsen inden for et 1 år efter sidste patient sidste forsøg.

## Etiske spørgsmål

Kun patienter, som underskriver informeret samtykke, indgår i projektet. Oplysninger om forsøgspersonerne beskyttes efter lov om behandling af personoplysninger og sundhedsloven. Projektet anmeldes til lokal kontakt person BBH, som derefter via paraply tilladelse giver besked til data tilsynet. Projektet sendes til sundhedsstyrelsen og videnskabsetisk komité. Patienterne kan til enhver tid kontakte projektansvarlige læge Marie Michelsen med henblik på yderligere spørgsmål om projektet. Patienter med mikrovaskulær sygdom (CFR<2.0) har i studier vist at have større risiko for kardiovaskulære hændelser end patienter med CFR>2. Projektet vil belyse mekanistiske effekter af ACE-hæmmer behandling og vil medvirke til forståelsen af om ACE-hæmmer kan forbedre mikrovaskulær funktion direkte og dermed forebygge vaskulær dysfunktion og senere sværere kardiovaskulær sygdom. Patienter vil blive behandlet i 6±1,5 måneder (50% med ramipril) for deres småkarssygdom. Risici og ulemper menes derfor at opvejes af de potentielle positive effekter af studiet.

Der er respekt for forsøgspersonernes fysiske og mentale integritet samt privatlivets fred og oplysningerne om forsøgspersonen beskyttes efter [lov om behandling af personoplysninger](https://www.retsinformation.dk/Forms/R0710.aspx?id=828) og [sundhedsloven](https://www.retsinformation.dk/forms/r0710.aspx?id=130455).

### Gennemførlighed

Forsøgspopulationen udgøres af patienter fra en allerede eksisterende kohorte af kvinder med angina pectoris uden stenoser påvist ved koronar arteriografi, iPower-studiet^1^. Der er 111 antal patienter som møder inklusions og eksklusion kriterier pr. 09.05.2014. Inklusion til iPower kohorten er kontinuerlig til start af ACIM-studiet således at denne population fortsat vil vokse inden inklusion til ACIM afsluttes. Der forudses derfor ingen problemer med rekvirering af forsøgspersoner til studiet.

## Reference liste

1. Prescott, E. et al. Improving diagnosis and treatment of women with angina pectoris and microvascular disease: the iPOWER study design and rationale. Am. Heart J. **167,** 452–458 (2014).

2. Camici, P. G. & Crea, F. Coronary Microvascular Dysfunction. N. Engl. J. Med. **356,** 830–840 (2007).

3. Cannon, R. O., 3rd. Microvascular angina and the continuing dilemma of chest pain with normal coronary angiograms. J. Am. Coll. Cardiol. **54,** 877–885 (2009).

4. Pepine, C. J. et al. Coronary microvascular reactivity to adenosine predicts adverse outcome in women evaluated for suspected ischemia results from the National Heart, Lung and Blood Institute WISE (Women’s Ischemia Syndrome Evaluation) study. J. Am. Coll. Cardiol. **55,** 2825–2832 (2010).

5. Britten, M. B., Zeiher, A. M. & Schächinger, V. Microvascular dysfunction in angiographically normal or mildly diseased coronary arteries predicts adverse cardiovascular long-term outcome. Coron. Artery Dis. **15,** 259–264 (2004).

6. Lee, D.-H. et al. Coronary flow reserve is a comprehensive indicator of cardiovascular risk factors in subjects with chest pain and normal coronary angiogram. Circ. J. Off. J. Jpn. Circ. Soc. **74,** 1405–1414 (2010).

7. Tuccillo, B. et al. Factors predicting coronary flow reserve impairment in patients evaluated for chest pain: an ultrasound study. J. Cardiovasc. Med. Hagerstown Md **9,** 251–255 (2008).

8. Chen, J.-W., Hsu, N.-W., Wu, T.-C., Lin, S.-J. & Chang, M.-S. Long-term angiotensin-converting enzyme inhibition reduces plasma asymmetric dimethylarginine and improves endothelial nitric oxide bioavailability and coronary microvascular function in patients with syndrome X. Am. J. Cardiol. **90,** 974–982 (2002).

9. Pauly, D. F. et al. In women with symptoms of cardiac ischemia, nonobstructive coronary arteries, and microvascular dysfunction, angiotensin-converting enzyme inhibition is associated with improved microvascular function: A double-blind randomized study from the National Heart, Lung and Blood Institute Women’s Ischemia Syndrome Evaluation (WISE). Am. Heart J. **162,** 678–684 (2011).

10. Schwartzkopff, B., Brehm, M., Mundhenke, M. & Strauer, B. E. Repair of coronary arterioles after treatment with perindopril in hypertensive heart disease. Hypertension **36,** 220–225 (2000).

11. Motz, W. & Strauer, B. E. Improvement of coronary flow reserve after long-term therapy with enalapril. Hypertension **27,** 1031–1038 (1996).

12. Neglia, D. et al. Perindopril and indapamide reverse coronary microvascular remodelling and improve flow in arterial hypertension. J. Hypertens. **29,** 364–372 (2011).

13. Akinboboye, O. O., Chou, R.-L. & Bergmann, S. R. Augmentation of myocardial blood flow in hypertensive heart disease by angiotensin antagonists: a comparison of lisinopril and losartan. J. Am. Coll. Cardiol. **40,** 703–709 (2002).

14. Masuda, D. et al. Evaluation of coronary blood flow reserve by 13N-NH3 positron emission computed tomography (PET) with dipyridamole in the treatment of hypertension with the ACE inhibitor (Cilazapril). Ann. Nucl. Med. **14,** 353–360 (2000).

15. Kawata, T. et al. Effect on coronary flow velocity reserve in patients with type 2 diabetes mellitus: comparison between angiotensin-converting enzyme inhibitor and angiotensin II type 1 receptor antagonist. Am. Heart J. **151,** 798.e9–15 (2006).

16. Rakugi, H., Wang, D. S., Dzau, V. J. & Pratt, R. E. Potential importance of tissue angiotensin-converting enzyme inhibition in preventing neointima formation. Circulation **90,** 449–455 (1994).

17. Heeneman, S., Sluimer, J. C. & Daemen, M. J. A. P. Angiotensin-converting enzyme and vascular remodeling. Circ. Res. **101,** 441–454 (2007).

18. Fox, K. M. & EURopean trial On reduction of cardiac events with Perindopril in stable coronary Artery disease Investigators. Efficacy of perindopril in reduction of cardiovascular events among patients with stable coronary artery disease: randomised, double-blind, placebo-controlled, multicentre trial (the EUROPA study). Lancet **362,** 782–788 (2003).

19. Yusuf, S. et al. Effects of an angiotensin-converting-enzyme inhibitor, ramipril, on cardiovascular events in high-risk patients. The Heart Outcomes Prevention Evaluation Study Investigators. N. Engl. J. Med. **342,** 145–153 (2000).

20. Pitt, B. et al. The QUinapril Ischemic Event Trial (QUIET): evaluation of chronic ACE inhibitor therapy in patients with ischemic heart disease and preserved left ventricular function. Am. J. Cardiol. **87,** 1058–1063 (2001).

21. Braunwald, E. et al. Angiotensin-converting-enzyme inhibition in stable coronary artery disease. N. Engl. J. Med. **351,** 2058–2068 (2004).

22. Pepine, C. J. et al. Effects of angiotensin-converting enzyme inhibition on transient ischemia: the Quinapril Anti-Ischemia and Symptoms of Angina Reduction (QUASAR) trial. J. Am. Coll. Cardiol. **42,** 2049–2059 (2003).

23. MacMahon, S. et al. Randomized, placebo-controlled trial of the angiotensin-converting enzyme inhibitor, ramipril, in patients with coronary or other occlusive arterial disease. PART-2 Collaborative Research Group. Prevention of Atherosclerosis with Ramipril. J. Am. Coll. Cardiol. **36,** 438–443 (2000).

24. Teo, K. K. et al. Long-term effects of cholesterol lowering and angiotensin-converting enzyme inhibition on coronary atherosclerosis: The Simvastatin/Enalapril Coronary Atherosclerosis Trial (SCAT). Circulation **102,** 1748–1754 (2000).

25. Skaug, E.-A., Madssen, E., Aspenes, S. T., Wisløff, U. & Ellingsen, O. Cardiovascular Risk Factors Have Larger Impact on Endothelial Function in Self-Reported Healthy Women than Men in the HUNT3 Fitness Study. PloS One **9,** e101371 (2014).

26. Hamburg, N. M. et al. Cross-sectional relations of digital vascular function to cardiovascular risk factors in the Framingham Heart Study. Circulation **117,** 2467–2474 (2008).

27. Shechter, M., Shechter, A., Koren-Morag, N., Feinberg, M. S. & Hiersch, L. Usefulness of brachial artery flow-mediated dilation to predict long-term cardiovascular events in subjects without heart disease. Am. J. Cardiol. **113,** 162–167 (2014).

28. Pizzi, C., Manfrini, O., Fontana, F. & Bugiardini, R. Angiotensin-converting enzyme inhibitors and 3-hydroxy-3-methylglutaryl coenzyme A reductase in cardiac Syndrome X: role of superoxide dismutase activity. Circulation **109,** 53–58 (2004).

29. Anderson, T. J., Elstein, E., Haber, H. & Charbonneau, F. Comparative study of ACE-inhibition, angiotensin II antagonism, and calcium channel blockade on flow-mediated vasodilation in patients with coronary disease (BANFF study). J. Am. Coll. Cardiol. **35,** 60–66 (2000).

30. Koh, K. K. et al. Mechanism by which quinapril improves vascular function in coronary artery disease. Am. J. Cardiol. **83,** 327–331 (1999).

31. Souza-Barbosa, L. A. et al. Endothelial vascular function in hypertensive patients after renin-angiotensin system blockade. J. Clin. Hypertens. Greenwich Conn **8,** 803–809; quiz 810–811 (2006).

32. Rahman, S. T. et al. The impact of lipoic acid on endothelial function and proteinuria in quinapril-treated diabetic patients with stage I hypertension: results from the QUALITY study. J. Cardiovasc. Pharmacol. Ther. **17,** 139–145 (2012).

33. Kovacs, I., Toth, J., Tarjan, J. & Koller, A. Correlation of flow mediated dilation with inflammatory markers in patients with impaired cardiac function. Beneficial effects of inhibition of ACE. Eur. J. Heart Fail. **8,** 451–459 (2006).

34. Ghiadoni, L. et al. Different effect of antihypertensive drugs on conduit artery endothelial function. Hypertension **41,** 1281–1286 (2003).

35. Morillas, P. et al. Circulating biomarkers of collagen metabolism in arterial hypertension: relevance of target organ damage. J. Hypertens. **31,** 1611–1617 (2013).

36. Laviades, C. et al. Abnormalities of the extracellular degradation of collagen type I in essential hypertension. Circulation **98,** 535–540 (1998).

37. Poulsen, S. H., Høst, N. B. & Egstrup, K. Long-term changes in collagen formation expressed by serum carboxyterminal propeptide of type-I procollagen and relation to left ventricular function after acute myocardial infarction. Cardiology **96,** 45–50 (2001).

38. Kosmala, W. et al. A randomized study of the beneficial effects of aldosterone antagonism on LV function, structure, and fibrosis markers in metabolic syndrome. JACC Cardiovasc. Imaging **4,** 1239–1249 (2011).

39. Claridge, M. W. et al. ACE inhibitors increase type III collagen synthesis: a potential explanation for reduction in acute vascular events by ACE inhibitors. Eur. J. Vasc. Endovasc. Surg. Off. J. Eur. Soc. Vasc. Surg. **28,** 67–70 (2004).

40. Carlsson, A. C. et al. Association between circulating endostatin, hypertension duration, and hypertensive target-organ damage. Hypertension **62,** 1146–1151 (2013).

41. Campbell, D. J. et al. Reduced microvascular density in non-ischemic myocardium of patients with recent non-ST-segment-elevation myocardial infarction. Int. J. Cardiol. **167,** 1027–1037 (2013).

42. Campbell, D. J. et al. Differences in myocardial structure and coronary microvasculature between men and women with coronary artery disease. Hypertension **57,** 186–192 (2011).

### Annex 1: medicinudlevering

**
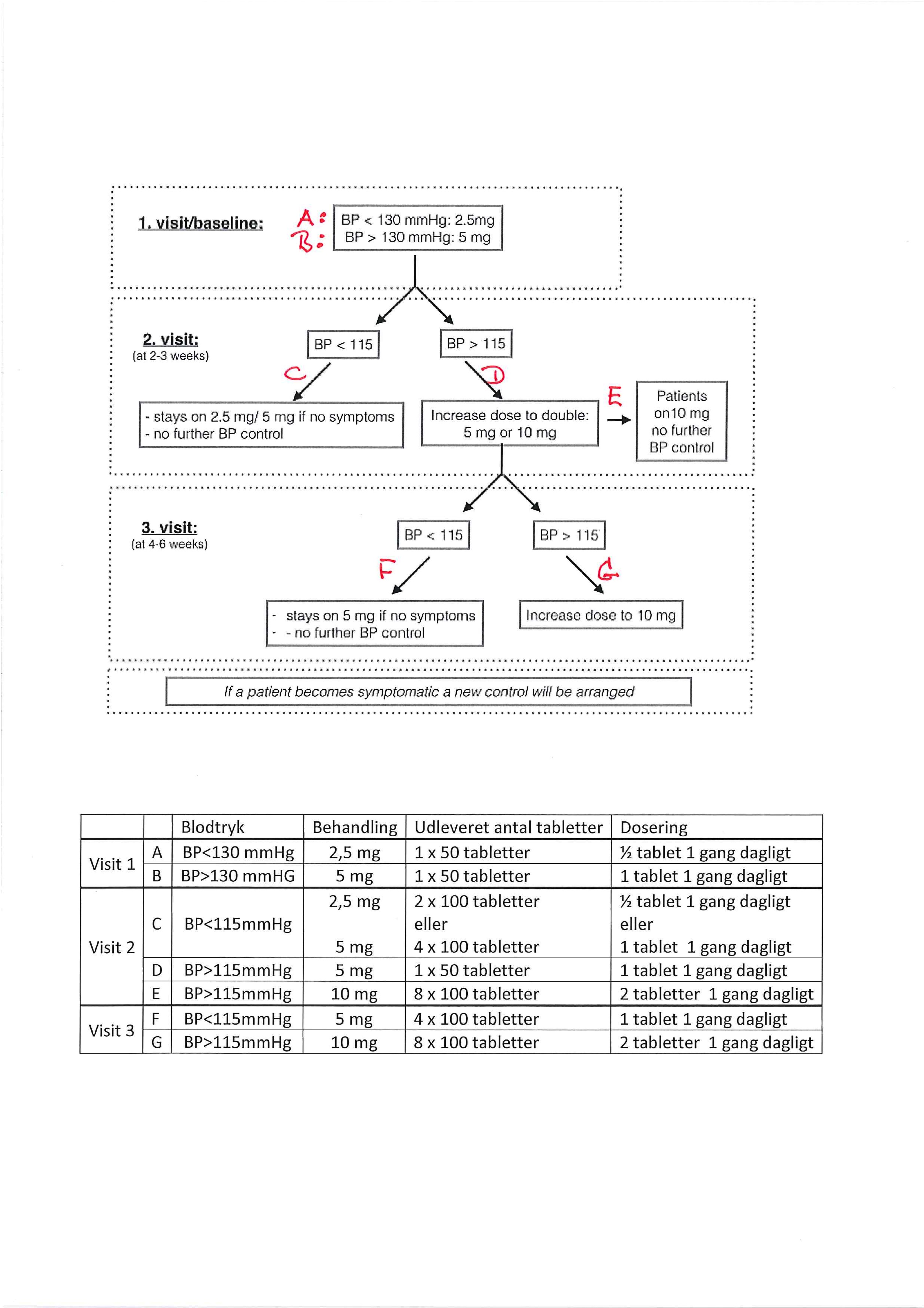
**
